# Zero-Threshold Optical Gain in Electrochemically Doped Nanoplatelets and the Physics Behind It

**DOI:** 10.1021/acsnano.2c07519

**Published:** 2022-10-18

**Authors:** Jaco J. Geuchies, Robbert Dijkhuizen, Marijn Koel, Gianluca Grimaldi, Indy du Fossé, Wiel H. Evers, Zeger Hens, Arjan J. Houtepen

**Affiliations:** † Optoelectronic Materials Section, Faculty of Applied Sciences, Delft University of Technology, Van der Maasweg 9, 2926 HZDelft, The Netherlands; ‡ Department of Chemistry and Center for Nano and Biophotonics, Ghent University, 9000Ghent, Belgium

**Keywords:** nanoplatelets, femtosecond transient absorption spectroscopy, electrochemistry, doping, optical gain

## Abstract

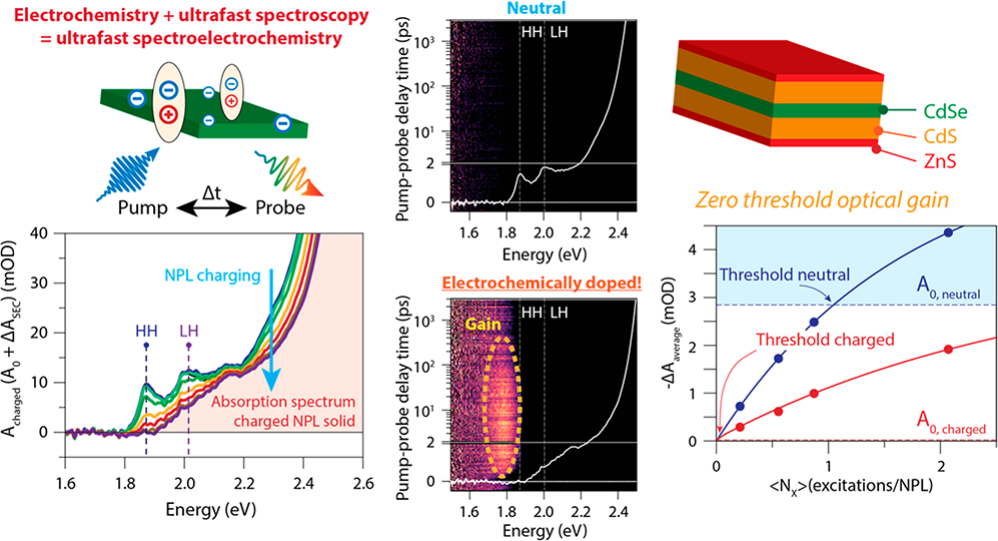

Colloidal nanoplatelets (NPLs) are promising materials for lasing applications. The properties are usually discussed in the framework of 2D materials, where strong excitonic effects dominate the optical properties near the band edge. At the same time, NPLs have finite lateral dimensions such that NPLs are not true extended 2D structures. Here we study the photophysics and gain properties of CdSe/CdS/ZnS core–shell–shell NPLs upon electrochemical n doping and optical excitation. Steady-state absorption and PL spectroscopy show that excitonic effects are weaker in core–shell–shell nanoplatelets due to the decreased exciton binding energy. Transient absorption studies reveal a gain threshold of only one excitation per nanoplatelet. Using electrochemical *n* doping, we observe the complete bleaching of the band edge exciton transitions. Combining electrochemical doping with transient absorption spectroscopy, we demonstrate that the gain threshold is fully removed over a broad spectral range and gain coefficients of several thousand cm^–1^ are obtained. These doped NPLs are the best performing colloidal nanomaterial gain medium reported to date, with the lowest gain threshold and broadest gain spectrum and gain coefficients that are 4 times higher than in n-doped colloidal quantum dots. The low exciton binding energy due to the CdS and ZnS shells, in combination with the relatively small lateral size of the NPLs, results in excited states that are effectively delocalized over the entire platelet. Core–shell NPLs are thus on the border between strong confinement in QDs and dominant Coulombic effects in 2D materials. We demonstrate that this limit is in effect ideal for optical gain and that it results in an optimal lateral size of the platelets where the gain threshold per nm^2^ is minimal.

## Introduction

Colloidal semiconductor nanomaterials have the potential to form efficient lasers. Solution processing allows facile integration into various device architectures, including conformal coating on patterned substrates.^1,2^ The size tunability due to quantum confinement translates into control over the gain spectrum and lasing color. The optimal gain material has a low gain threshold (low threshold carrier density), a long gain lifetime (the time during which there is population inversion and the absorption of the material stays negative), and a high gain coefficient (the fractional increase of radiant energy of an incident photon beam per unit length). Most studies in this field have focused on CdSe quantum dots (QDs),^3,4^ where the underlying photophysics are broadly understood.^5^ Recently it was shown by various groups, including ours,^6−9^ that n doping allows the decrease of the threshold for optical gain. At the same time, of the various colloidal nanomaterials that have been investigated, 2D nanoplatelets (NPLs) of II–VI semiconductors have stood out with the lowest gain threshold and the highest gain coefficients.^10−13^ This provokes the suggestion that n-doped nanoplatelets could be the ultimate colloidal gain material. However, substantiating this hypothesis with a theoretical description of gain in doped NPLs is complicated by the fact that the photophysics in 2D NPLs is quite different from that in 0D QDs.

In QDs, the optical properties are dominated by quantum confinement, which is stronger than Coulomb interactions (the so-called “strong confinement regime”). The development of gain is dominated by state filling, and Coulomb interactions between carriers act only as a perturbation. Excitation of higher energy levels is followed by relaxation to the 1S(e) and 1S_3/2_(h) levels, making QDs a three-level lasing system.^3^ For II–VI and III–V QDs this results in a gain threshold of 4/3 excitations per QD (∼1.54 excitations per QD when including Poisson excitation statistics),^9,14,15^ and consequently, Auger recombination (AR) of multiple excitons is the main factor that prevents the buildup of gain using continuous wave excitation. Therefore, much work has focused on understanding and slowing down AR.^16−21^ One way to decrease the gain threshold to below a single exciton per QD is by electronic doping. This changes the QDs from a three- to a four-level lasing system.^6,7,9^

In contrast, the optical properties in NPLs are usually considered to be those of 2D semiconductors, dominated by excitonic effects, due to strong Coulomb interactions between electrons and holes. Optical excitation or charging of NPLs with electrons results in state filling and a bleach of the excitonic and free carrier absorption^22^ but in addition results in screening of both the electron–hole Coulomb and exchange interactions by the additional carriers. Such screening is expected to decrease the exciton binding energy, which decreases the oscillator strength of the exciton transition and leads to lifetime broadening due to increased scattering and increases exchange interactions.^23,24^ As the screening increases with exciton or electron density, a Mott transition to an electron–hole plasma may take place.^25^

In a hydrogenic model, the 2D exciton binding energy is 4 times the bulk (3D) exciton binding energy. This increase is further enhanced by dielectric confinement in colloidal NPLs. As a result, the exciton binding energy in CdSe NPLs is typically ∼200 meV (whereas the bulk exciton binding energy is 15 meV)^12^ and excitons are extremely robust at room temperature. The high exciton binding energy corresponds to very small exciton Bohr radii,^24,26^ which in turn means that state filling of the exciton transition is minor, even at high excitation densities.^13^ Complete bleaching of the exciton transition has thus not been observed in CdSe NPLs, and gain by free carriers or exciton molecules strongly suffers from competing exciton absorption.

Here we investigate the development of gain in optically excited and electrochemically n-doped CdSe/CdS/ZnS core–shell–shell (CSS) NPLs. The use of core–shell–shell NPLs is necessary for stable and reproducible electrochemical doping but also results in a much smaller exciton binding energy. The core–shell structure effectively eliminates dielectric confinement such that the exciton binding energy is of the order of 40 meV. These excitons are much larger than in core only CdSe NPLs and thus more sensitive to screening and state filling. We demonstrate that we can fully bleach the optical transitions either by photoexcitation or by electrochemical n doping. Surprisingly, in undoped NPLs, we find a gain threshold of only one excitation per nanoplatelet. We combine n doping with photoexcitation in ultrafast spectroelectrochemical measurements to probe the development of optical gain in doped NPLs. We find the lowest gain threshold reported for colloidal nanomaterials and gain spectra with a broad bandwidth. In addition, the gain coefficients are 3–4 times higher than in the best n-doped QD films and show no sign of saturation. This demonstrates that doped NPLs are indeed an extremely promising gain medium. The results show that the low exciton binding energy due to the CdS and ZnS shell, in combination with the relatively small lateral size of the NPLs, results in excited states that are effectively delocalized over the entire platelet, which consequently behave as a particle in a box. These core–shell nanoplatelets are on the border between strong confinement in QDs and dominant Coulombic effects resulting in excitonic behavior in 2D materials. We demonstrate that this limit is in effect ideal for optical gain and that it results in an optimal lateral size of ∼500 nm^2^ of the platelets where the gain threshold per nm^2^ is minimal.

The paper is organized as follows: we start by discussing the steady state optical properties of CdSe/CdS/ZnS NPLs of varying shell thickness. We then controllably add excitons, electrons, or both to investigate the effects of state filling and screening. Finally, we quantify optical gain in films of n-doped NPLs and demonstrate that the gain threshold vanishes at the highest doping density.

## Results and Discussion

### Excitons in Core–Shell(−Shell) NPLs?

**Figure 1:**
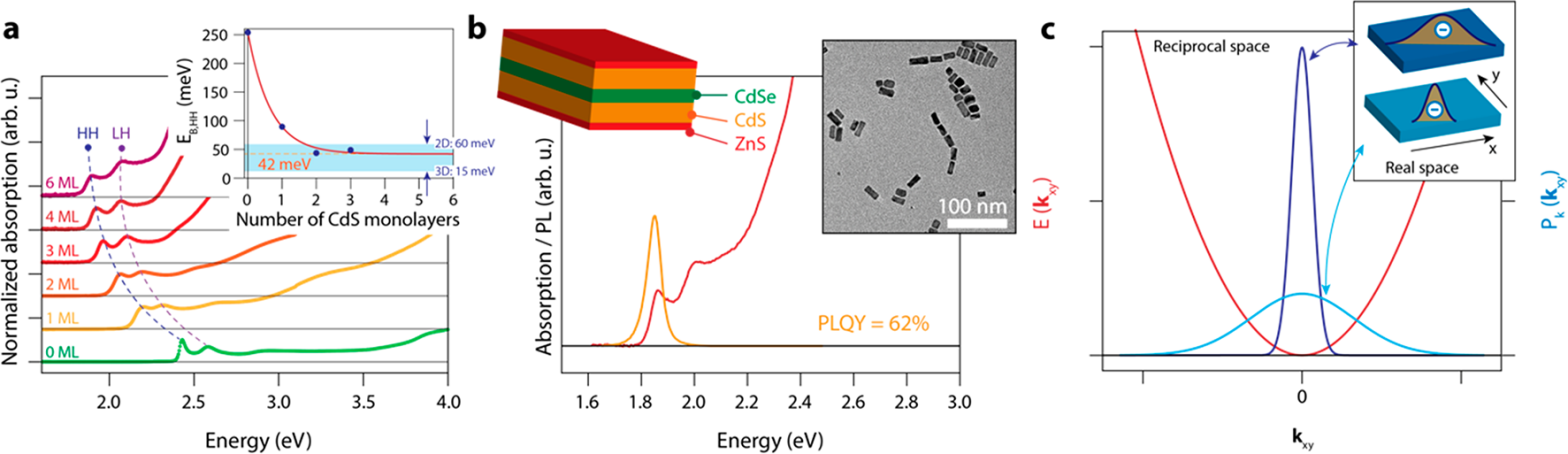
Steady-state optical properties of CdSe/CdS(/ZnS) core–shell(−shell) nanoplatelets. (a) Optical absorption of CdSe-based NPLs with increasing shell thickness. The inset shows the estimated HH exciton binding energy as a function of shell thickness. For the main NPLs studied throughout this work (6 MLs of CdS, 2 MLs of ZnS), we estimate an exciton binding energy of 42 meV. (b) Absorption and photoluminescence spectra of a dispersion of CdSe/CdS/ZnS NPLs in hexane. The inset shows a representative TEM micrograph. (c) Schematic of the dispersion relation *E*(**k**_*xy*_) versus in-plane wavevector **k**_*xy*_ and probability amplitude *P*_k_(**k**_*xy*_) of the electron wave function in real (inset) and reciprocal space.

We synthesized zinc-blende CdSe NPLs which emit around 510 nm via procedures outlined in the methods section. We have grown CdS and ZnS layers around the CdSe core NPLs by a continuous injection method^2,27,28^ in order to enhance their photoluminescence quantum yield (PLQY) and their photo- and electrochemical^29^ stability. In Figure 1a, we present the absorption spectra for the CdSe/CdS NPLs with shell thicknesses varying from one to six monolayers. This results in a gradual redshift of the absorption spectrum with increasing CdS shell thickness. Moreover, the intensity ratio between the heavy hole (HH) and light hole (LH) absorption gradually decreases. This is not due to a relative increase in oscillator strength of the LH transition but rather due to a decrease in exciton binding energy, a consequence of the decreased dielectric contrast between the optically active CdSe core and its surroundings (ligands vs CdS) when a shell is grown, which results in free carrier absorption that overlaps with the LH line.

Using methods outlined in literature,^12,23,30^ we decompose the absorption spectra of the core-only and core–shell NPLs in excitonic and free-carrier absorption to get an estimate of the HH-exciton binding energy. The full analysis is shown in the Supporting Information, and the results are shown as the inset of Figure 1a. The HH exciton binding energy (*E*_B,HH_) of 254 meV in the core-only CdSe NPLs decreases to 89 meV upon growing one monolayer of CdS on the NPL surface. For the core–shell–shell CdSe/CdS/ZnS NPLs used throughout this study, which are coated with six monolayers of CdS and two monolayers of ZnS, we estimate *E*_B,HH_ to be around 42 meV, which is in between the bulk (15 meV)^31^ and the 2D limit (4 times bulk = 60 meV) exciton binding energies^32^ (see Supporting Information Section S2). In the 2D hydrogenic model a binding energy of 42 meV corresponds to a 2D exciton Bohr radius of *a*_B,2D_ = 3.2 nm, compared to *a*_B,2D_ = 0.53 nm for the core only NPLs. The final CdSe/CdS/ZnS NPLs, with 6 CdS and 2 ZnS monolayers, presented in Figure 1b, have a PLQY of 62% and have lateral dimensions of 24.8 ± 1.6 nm by 9.9 ± 0.6 nm. Especially the ZnS shell enhances the electrochemical stability, similar to observations we made on CdSe/CdS/ZnS QDs,^29,33,34^ as shown in the Supporting Information (Section S3).

To check whether, due to the lower *E*_B,HH_, excitons still form in these CSS NPLs, we performed temperature dependent absorption and PL measurements down to 13 K, presented in Figure S13. In contrast to more traditional quantum wells, such as GaAs ^35^ (*E*_b,HH_ ≤ 10 meV ^36^), there is no distinct sign of a crossover from excitonic to free carrier transitions.^37^ This could either mean that *E*_B,HH_ is still so high that the optical features at room temperature are still dominated by excitons, or it could mean that the electron and hole wave functions are fully delocalized over the NPLs and the optical features are the result of quantum confinement; *vide infra.*

### Optical Gain in CSS NPLs

Next, we analyze the effect of photoexciting CSS NPLs dispersed in hexane with fs transient absorption (TA) spectroscopy. We measure the change in absorption Δ*A*_TA_ upon excitation with 400 nm pump pulses as a function of time delay and excitation density. Measuring the fluence dependence of the bleach amplitude allows us to obtain the absorption cross section by assuming Poisson excitation statistics and Auger recombination of multiexcitons.^9^ Following this approach, we determined the cross-section at 400 nm, σ_400nm_, to be 3.3 × 10^–14^ cm^2^. As outlined in the Supporting Information (Section S5), we used multiple alternative procedures to verify this cross section, as it is important to determine the excitation density per NPL ⟨*N_X_*⟩ = *J*σ_400nm_, where *J* is the photon fluence per cm^2^ per laser pulse.

**Figure 2:**
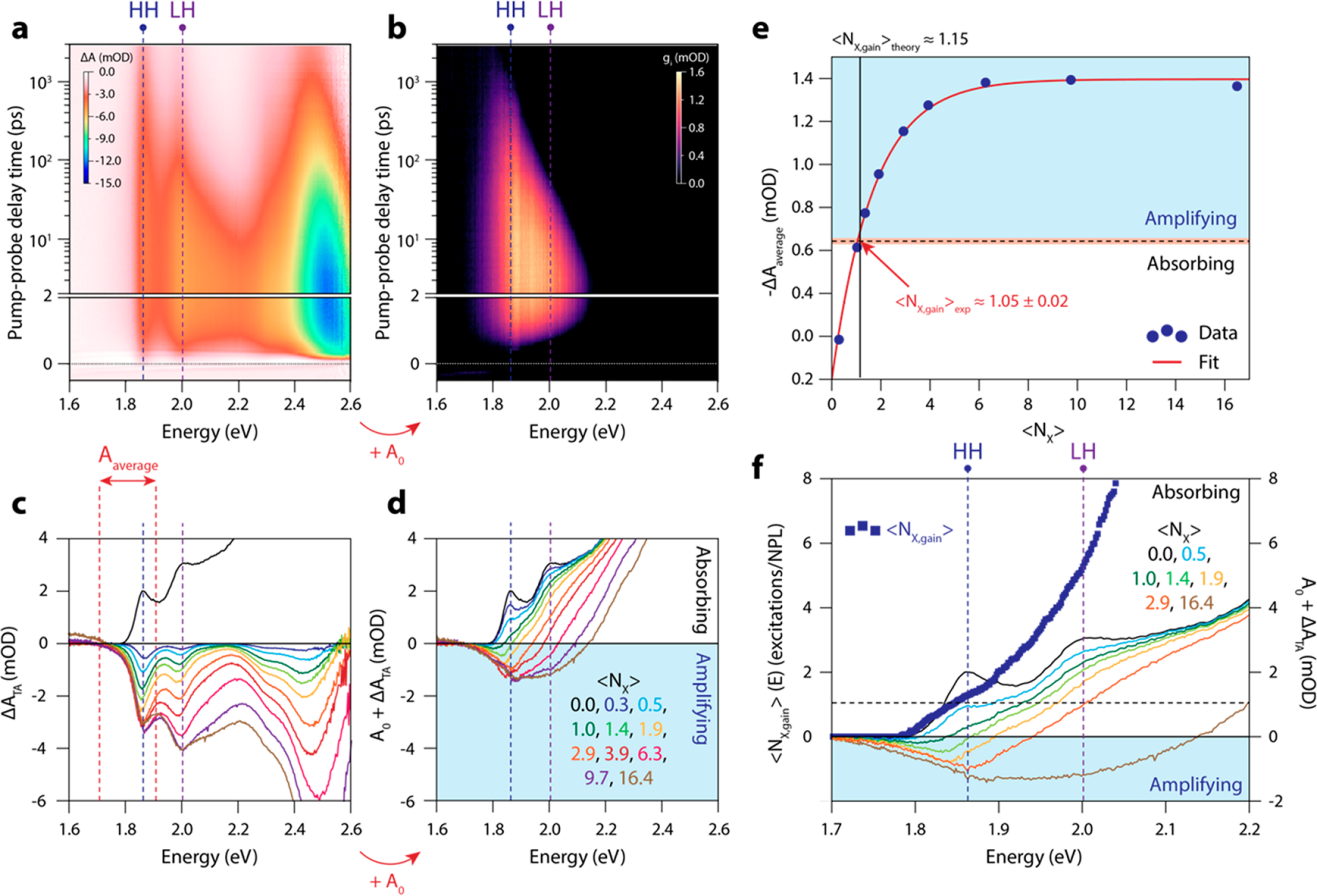
Optical gain characteristics of isolated CSS NPLs in solution after 400 nm pump. (a) Transient absorption map for ⟨*N_X_*⟩ = 16.4. (b) Optical gain map of (a), obtained by adding the steady-state absorption to the TA signal (*A*_0_ + Δ*A*_TA_). (c) Spectral slices of the TA map at 5 ps pump–probe delay time, for increasing excitation density. (d) Gain spectra at 5 ps for increasing excitation density. (e) Determination of the spectrally averaged (between 1.7 and 1.9 eV) optical gain threshold for the HH transition, ⟨*N*_*X*,gain_⟩ = 1.05 ± 0.02 excitations/NPL. (f) Energy-dependent threshold for optical gain, by repeating the analysis shown in (e) for each energy. On the red side of the HH transition, less than one excitation per NPL will lead to optical gain.

Figure 2a shows a TA map for an excitation density of ⟨*N_X_*⟩ = 16.4. The HH and LH bleach maxima are indicated with dashed lines. Figure 2b shows an optical gain map, acquired by adding the steady-state absorption *A*_0_ to the Δ*A*_TA_ signal to obtain the excited-state absorption *A*′. Positive absorption is color-coded black, whereas negative absorption, i.e., optical gain, is color-coded according to the optical density. Note that in the gain map, the HH and LH transitions are strongly broadened, and the lifetime of the gain signal is longest on the red side.

Spectral slices through the TA and optical gain data are presented in Figure 2c and Figure 2d, respectively, for a pump–probe delay time of 10 ps. While clear bleach features of the HH and LH exciton lines are observed, in the optical gain spectra, there is no sign of gain from the distinct transitions. Instead, it appears that optical gain develops first on the red side at low fluence, consistent with work on core-only NPLs, that attribute this to gain from biexciton molecules.^12^ At higher fluence, the gain spectrum broadens, spanning both the HH and LH transition and featuring a spectral width of roughly 500 meV, similar to the widest gain bandwidth observed on giant core–shell CdSe/CdS QDs.^38^ Strikingly, we observe a complete bleach of the HH and LH exciton absorption features at low excitation densities (⟨*N_X_*⟩ ∼ 1), in stark contrast to work on core-only CdSe NPLs,^13^ whereupon creating 112 excitations on average per NPL, the HH and LH transitions were still prominently visible in the excited-state absorption spectrum.

To quantitatively capture the photophysics for optical gain and determine ⟨*N*_*X*,gain_⟩ for the HH transition, we spectrally average the steady-state absorption and bleach amplitude (between the dashed red lines in Figure 2c) for each fluence. This spectral averaging corrects for spectral shifts and reveals the total change in the absorption strength of the HH transition, due to state filling and stimulated emission or a reduction in the oscillator strength. As shown in Figure 2e, we find that ⟨*N*_*X*,gain_⟩ = 1.05 ± 0.02, a figure we will come back to shortly, which is equivalent to a 2D exciton density of 4 × 10^11^ cm^–2^. We note that this gain threshold is an average; the absorption cross section depends on the orientation of the NPLs, and hence there will be a distribution of excitation densities over the orientations in the NPL ensemble. The gain threshold of roughly one per NPL should be interpreted not as exact but as an indication that only a few excitations per NPL are required to achieve optical gain. Furthermore, we determined the gain threshold for all probe energies to obtain a so-called gain threshold spectrum *N*_*X*,gain_(*E*), which is shown as the blue solid squares in Figure 2f. For energies below the HH transition, we obtain optical gain for vanishingly low excitation densities in a spectral range up to 1.8 eV, where there is significant light amplification. The distinction between spectrally averaged and single wavelength gain thresholds is important^9,39^ although often neglected in the literature.

To understand what it means when the spectrally averaged gain threshold in these CSS NPLs is only ∼1, we consider the effect of state filling on excitons in 2D materials. The exciton state can be written as a linear combination of free carrier states, as shown schematically in Figure 1c. To form a small, localized exciton in real space requires the combination of many free carrier states with different momenta. The extension of the internal wave function in real space and momentum space is related via the Heisenberg uncertainty relation. Optical excitation or electronic doping leads to partial occupation of some free carrier states that are now no longer available for the formation of new excitons. This causes a decrease of the exciton absorption, an effect that is called state filling. In the case of a large exciton binding energy and a correspondingly small exciton Bohr radius, the number of *k*-states that contribute to the exciton wave function is large, and the bleach of a single free carrier (occupying a state near *k* = 0) is small. For a smaller binding energy this bleach will be larger. The low-gain threshold, where only approximately one excitation per NPL is required to achieve transparency, demonstrates that the excited-state internal wave function is dominated by one or few electron and hole free carrier states only. This implies that the average electron–hole interdistance is comparable to the size of the NPL, or *mutatis mutandum*, and that the Coulomb interaction is not the dominant factor in controlling the spatial extension of the internal wave function.

Due to the large exciton size, the wave function of the excited state will approach that of a particle in a box. If we calculate the sum of the confinement energy of electrons and holes in the lateral dimensions of the NPLs using a simple rectangular particle in a box model, we find a value of 49 meV (see Supporting Information Section S6 for details), almost the same as the exciton binding energy. This suggests that these NPLs are on the border between weak and strong confinement, i.e., on the border between single carriers and excitons. We can further estimate that the difference in energy of the electrons between the first and second quantized level in the longest dimension of the NPLs is ∼20 meV. This implies that at room temperature, most of the photoexcited electrons occupy the lowest quantized energy level.

For a truly extended 2D system the lateral size is not relevant for the gain properties. However, the NPLs investigated here are not extended 2D systems. An interesting question is how, in this limit, the lateral size influences the gain properties and whether an optimum lateral size exists. This optimum should be the size where the gain threshold density, measured in excitations per nm^2^, is lowest, as this corresponds to a medium that can show gain at the lowest photon fluence (in J cm^–2^ s^–1^ for continuous wave excitation).

To this end, we theoretically treat the NPL as a particle-in-a-box. We neglect excitonic effects and assume the energy levels of a particle-in-a-box with dimension given by the thickness of the NPLs, as well as the two lateral dimensions. The details are given in the Supporting Information Section S6. Further, we assume a quasi-Fermi–Dirac distribution in the conduction and valence band and compute the excitation density in terms of the number of excitations per nm^2^ that are required to achieve transparency. Figure S15 shows how the threshold density depends on the lateral sizes. A minimum is found for square platelets with lateral sizes of 23 nm, where the threshold density is around 0.012 excitations per nm^2^. Reducing the lateral area of a NPL increases the threshold excitation density (in excitations/nm^2^),

For small lateral sizes, a similar number of excitations per NPL (around 1) is required. Hence, reducing the lateral area of the NPLs increases the threshold excitation density in excitations/nm^2^. For larger lateral sizes, the energy spacing in both the conduction and valence band becomes so small that significant thermal population of higher levels occurs. This leads to an increase of the gain threshold per NPLs. The simulations show that the latter effect becomes dominant such that an optimum lateral size exists around 23 × 23 nm^2^, where the threshold density is minimal. The optimum platelet is square, since the energy spacing scales as the length^–2^ so that for a given area, the thermal population of higher levels is minimal if the two lateral lengths are the same. Importantly this shows that in the absence of excitonic effects, relatively small NPLs are better gain materials than extended 2D systems of the same thickness. The model can be extended to other semiconductor materials by changing the electron and hole effective masses.

### Electrochemical n Doping of CSS NPL Solids

**Figure 3:**
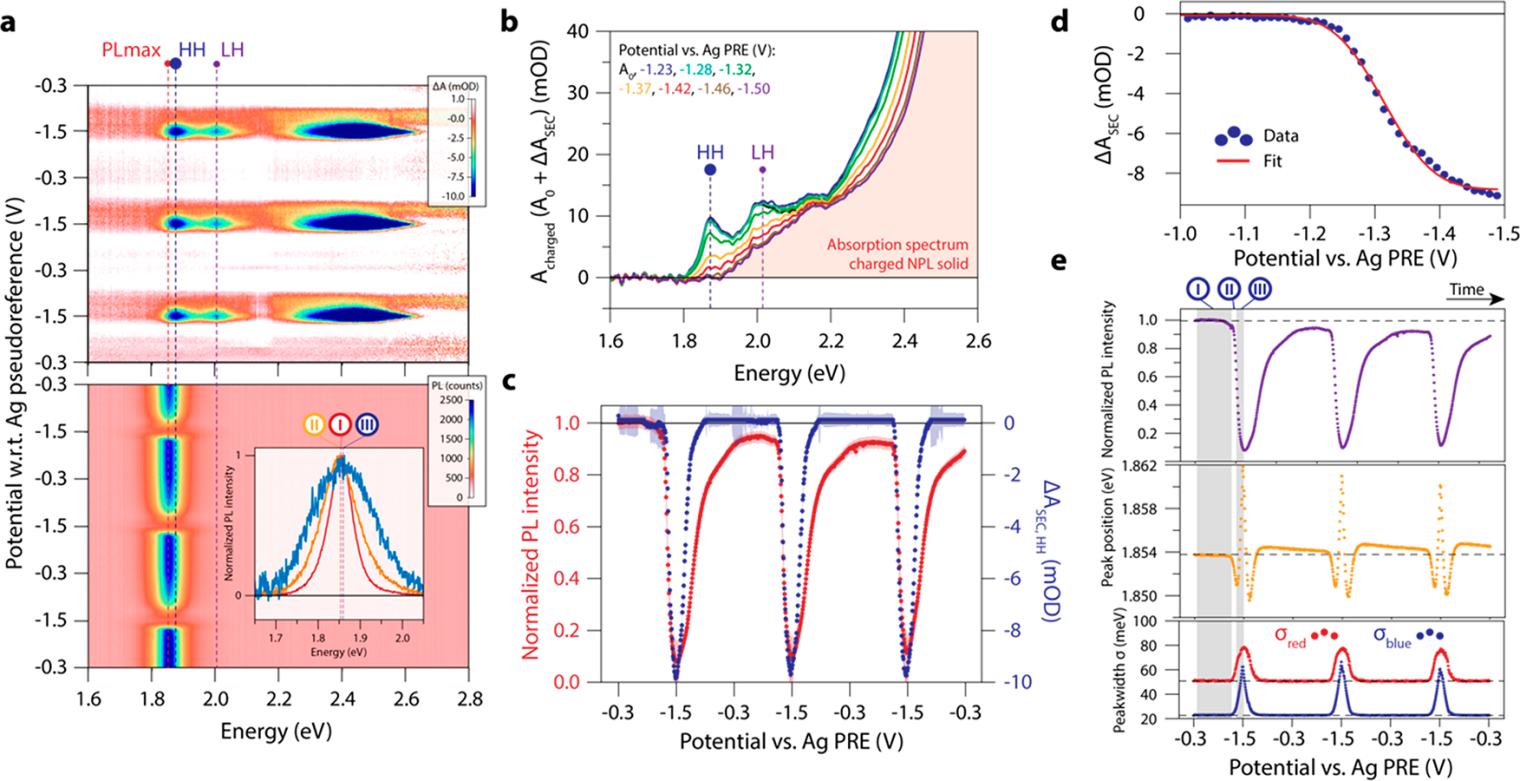
Spectroelectrochemistry on a CSS NPL film. (a) Absorption (top) and photoluminescence (bottom) SEC of a CSS NPL film. The inset at the bottom SEC-PL map shows individual PL spectra for the neutral film (−0.3 V, red line) at −1.32 V (most redshifted, yellow line) and −1.5 V (blue line). (b) Absorption spectra at different potentials. The distinct excitonic transitions (HH and LH) collapse and a broad, featureless absorption remains when charging the NPLs at −1.5 V vs the Ag PRE. (c) Normalized absorption bleach of the HH transition (blue) and PL intensity (red) during three potential sweeps. Note that the PL recovery is slower than the quenching; 5 min after the experiment, the original PL intensity is fully retrieved. (d) Absorption bleach amplitude vs electrochemical potential. The red line is a fit to a sigmoidal function. (e) Fitted peak amplitude, peak position, and standard deviation (on the red and blue side of the PL maximum) of the PL peak as a function of electrochemical potential.

To decrease the gain threshold further, we electrochemically charged the NPLs with electrons. We used spectroelectrochemistry (SEC) to determine the reproducibility and efficiency of electron injection.^40^ NPL films on ITO were prepared by consecutive dipcoating steps, as outlined in the Supporting Information. Figure 3a shows the absorption (top) and photoluminescence (PL, bottom) SEC data as a function of potential between the NPLs-on-ITO working electrode vs a Ag pseudoreference (PRE) electrode (with a calibrated potential of +4.35 V vs vacuum; see Supporting Information Figure S12). Upon decreasing the potential, the Fermi level of the ITO working electrode is raised and electrons are injected into the NPL solid. This is clearly demonstrated by the absorption bleach of several transitions, most notably the HH and LH transitions. The reversibility of charging and discharging is shown by sweeping the potential three times between the open circuit voltage (*V*_OC_ = −0.3 V) and −1.5 V. Note that core-only NPLs and CdSe/CdS core crown NPLs can also be charged but the maximum achievable doping density (judged from the absorption bleach) was much smaller, and the PL quenches irreversibly (see Supporting Information). This is indicative of permanent electrochemical changes on the surface of the NPLs, likely related to the partial reduction of surface Cd ions.^41,42^ In contrast, using CdSe/CdS/ZnS NPLs, we can controllably and reproducibly add electrons via electrochemical doping.

In Figure 3b we show absorption spectra at different potentials, obtained by adding the change in absorption due to electrochemically injected electrons, Δ*A*_SEC_, to the steady-state absorption of the film (Δ*A*_SEC_ + *A*_0_). We observe that the absorption at the HH transition is fully bleached, whereas at the energy of the LH transition, a broad and featureless absorption band with no signs of distinct excitonic transitions remains. We attribute this residual absorption to free-carrier absorption, whose onset overlaps with the LH transition.

In Figure 3c we compare the changes in the film absorption and PL as a function of the electrochemical potential. At around −1.25 V we observe that both the excitonic absorption bleaches and the PL quenches. This shows that electrons are injected into the conduction band; state filling decreases the absorption, and Auger recombination decreases the PL efficiency. The fact that the PL and absorption decrease at the same potential is an indication that the films are relatively trap-free and stable, since traps or electrochemical surface reactions often lead to PL changes when the Fermi level is still in the bandgap.^29,43^ The PL amplitude drops slightly over the course of consecutive potential cycles but is restored to its initial intensity within 5 min after the end of the experiment, suggesting that a small amount of charge is stored in relatively shallow traps.

To quantify both the absorption and PL SEC data in more detail, we fitted two Gaussian bleach functions to the absorption changes, and an asymmetric Gaussian function (with a different width on the red and blue side) to the PL data; the fitted parameters are presented in Figure 3d,e. The onset of the absorption bleach lies at around −1.25 V vs the Ag PRE (corresponding to −3.1 eV below vacuum), and a complete bleach of the exciton absorption is observed at around −1.40 V. The shape of Δ*A*(*V*) is well-described by an error function with a standard deviation of 60 mV. The PL data show that upon electron injection, the PL energy first redshifts by 3 meV, possibly due to shakeup processes,^44^ after which it blueshifts by 11 meV, likely to recombination of injected electrons in higher states in the conduction band. Moreover, the PL also broadens significantly. The broadening is stronger on the blue side of the PL spectrum than on the red side.

The electrochemical results show that we can completely bleach the HH exciton absorption by injecting electrons into the NPLs. We can estimate to what energy the Fermi level needs to be raised above the band edge (a value we term Δ*E*_tot_) to cause a complete bleach of the exciton absorption due to state filling based on a simple Heisenberg model (outlined in the Supporting Information, Section S7). The rationale is that from the exciton Bohr radius, the electron momentum can be estimated via the Heisenberg relation. When all states up to this momentum, which corresponds to  via the electron dispersion, are occupied, this causes a complete bleach due to state filling. As shown in the Supporting Information one can derive that
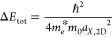
1

If we use the previously estimated 2D Bohr radius of 3.2 nm, we find that Δ*E*_tot_ should equal 14 meV, which is much less than the ∼120 mV that is needed to bleach the exciton line experimentally (Figure 3d). This observation suggests that state-filling alone should more quickly bleach the excitonic transitions than we observe. A possible explanation is that the potential change in the electrochemical experiment does not correspond one-to-one to the change of the Fermi level. The applied potential may drop partially over the ITO/NPL interface (as desired) and partially over the NPL/electrolyte interface. The latter part will not lead to a change in the Fermi level. In addition, possible side reactions, such as the reduction of molecular oxygen,^45^ could decrease the actual charge density, and hence the Fermi level. In this case the system is in a steady state rather than in a true equilibrium and the potential difference that is applied is larger than the change in the Fermi level.

### Ultrafast Spectroelectrochemistry

**Figure 4:**
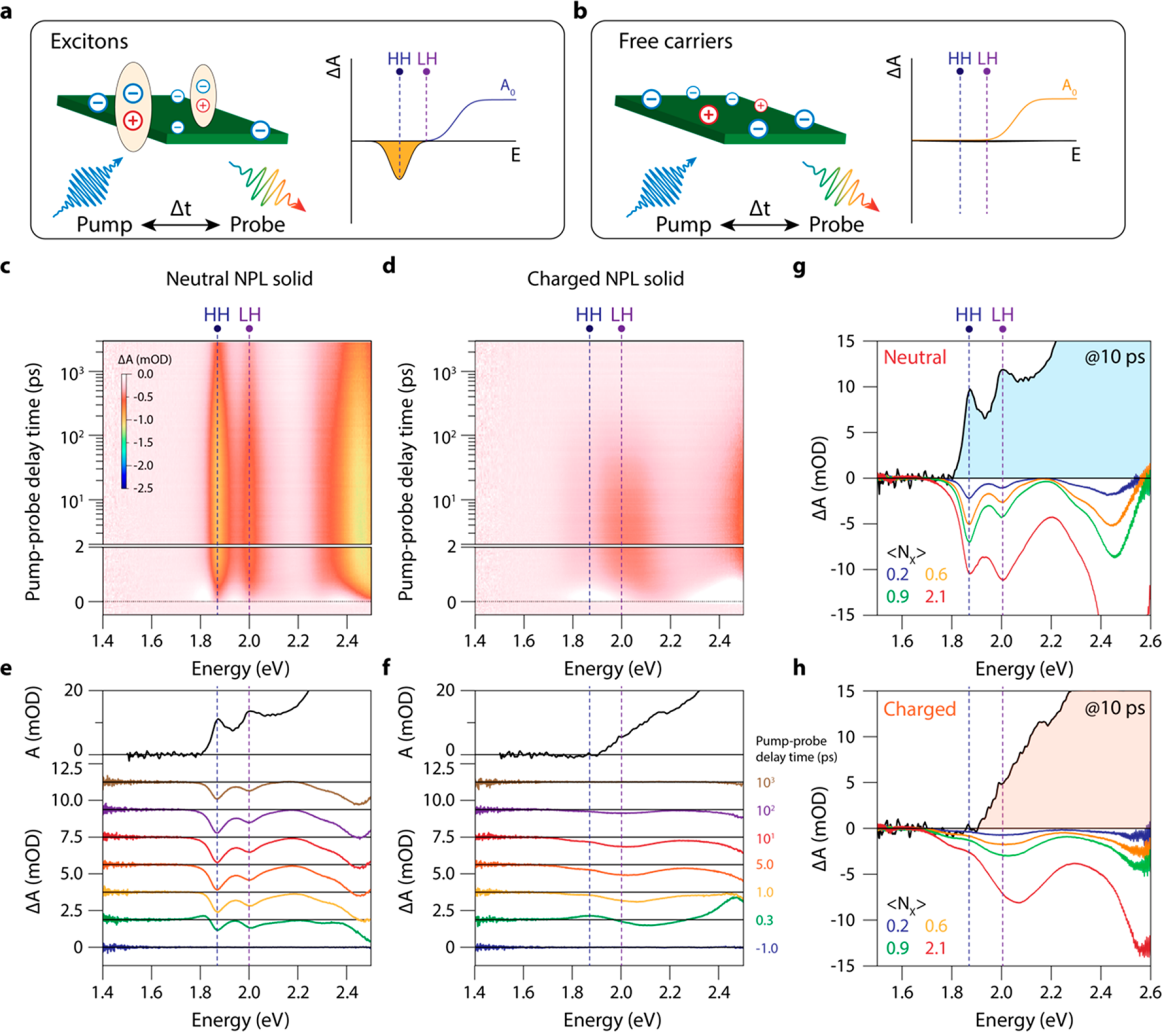
TA of the neutral and fully charged (−1.5 V vs Ag PRE) film after excitation at 400 nm (3.1 eV). (a) Schematic of the expected TA spectrum for doped NPLs where excitons are formed. This should result in narrow bleach features, resulting from SE from the excitons. (b) Schematic of the expected TA spectrum for doped NPLs where only free charges form. Here, the absence of SE from excitons should result in the absence of narrow bleach features. (c) TA map of the neutral NPL film, with ⟨*N_X_*⟩ = 0.2. A clear bleach of both the HH and LH excitons is observed. (d) TA map of the charged NPL film, with ⟨*N_X_*⟩ = 0.2. The distinct excitonic bleaches (HH and LH) disappear, and we observe SE from the HH exciton and bleaching/SE of the free-carrier transitions. (e) Spectral slices through the TA data of the neutral NPL film in (c) at different pump–probe delay times. (f) Spectral slices through the TA data of the charged NPL film in (d) at different pump–probe delay times. The distinct excitonic bleaches are replaced by broader features. (g) Δ*A*_TA_ spectra of the neutral NPL solid at 10 ps pump–probe delay time. Upon increasing the excitation density, the HH transition is fully bleached (Δ*A*_TA_ > *A*_0_), and narrow bleach features of the excitonic transitions are observed. (h) Δ*A*_TA_ spectra of the charged NPL solid at 10 ps pump–probe delay time. Already at low excitation densities, the spectra look different from the neutral NPL solid; the features are shifted and broadened, and the bleach amplitude is significantly decreased.

The TA and the SEC results presented above show that the excitonic absorption can fully bleach upon photoexcitation or electrochemical n doping. The resulting optical features appear broad and resemble free carriers. An open question is whether state filling alone is responsible for the disappearance of the exciton absorption, or whether Coulomb screening and enhanced electron–hole exchange interactions in the photoexcited or n-doped samples are also partially responsible for these observations. To test this, we combined electrochemical doping with fsTA measurement. We consider that if *only* state filling is responsible for the bleach of the excitonic transitions upon n doping, photoexcitation could still result in the formation of excitons. Their stimulated emission should show up as narrow features in fsTA measurements on doped films, with a spectral shape similar to the original exciton absorption, as depicted in Figure 4a. If, however, due to screening and increased exchange interactions, only free carriers remain in doped films, such sharp SE signal will not be observed, as depicted in Figure 4b.

The fsTA experiments are shown in Figure 4, using a pump wavelength of 400 nm, on a neutral (Figure 4c) and charged (at −1.5 V vs PRE, Figure 4d) NPL film. These TA maps were measured in the linear excitation density regime (⟨*N_X_*⟩ = 0.2). The neutral (c, e) and charged (d, f) films show strong differences in their TA responses. The HH (1.87 eV) and LH (2.0 eV) transitions appear as sharp and narrow bleach features in the neutral film with a lifetime that exceeds the pump–probe time delay window of our setup (3 ns). For the charged film (Figure 4d,f) we observe two broadened bleach features near the band-edge; a weak, broad bleach that is redshifted from the neutral HH transition by 90 meV and a second bleach that has its center at the LH peak position. The bleach feature at 2.55 eV in the doped NPL film is likely related to a transition in the CdS shell, as is also seen around 2.5 eV for the neutral NPLs. Compared to the neutral film, where the lifetime of the bleach features extends beyond the time window of our setup (3 ns), the bleach features in the charged film decay within ∼500 ps, as a result of efficient Auger recombination in the charged film. Furthermore, the absorption bleach at higher energy (>2.4 eV) is much weaker, and blueshifted, compared to the neutral film.

At short pump–probe delay times (<1 ps), we also observe differences between the charged and neutral NPL film. For the neutral film, in addition to an absorption bleach, there is some induced absorption (IA) on the red side of the HH and LH/free carrier transitions. This is often ascribed to bandgap renormalization and/or hot-carrier effects that result in a spectral shift of the absorption transitions (see Supporting Information Section S8 for more details). In contrast, for the charged film, there is no HH absorption in the ground state, so we do not observe a redshift of this feature. In contrast we observe induced absorption at the HH energy, probably as a result of a transient redshift of free carrier absorption. Negative Δ*A* of the HH transition appears after 1 ps in the charged film. Negative Δ*A* necessarily comes from stimulated emission (since there is no absorption at this energy in the ground state of the charged NPLs), and the onset of SE at the HH energy reflects the cooling of holes from the LH to the HH band.

Fluence dependent measurements (from ⟨*N_X_*⟩ = 0.2 to 2.1) are shown for the neutral and charged NPL solid in Figure 4g and Figure 4h, respectively. Narrow bleach features are observed for all excitation densities in the neutral film. There is no significant broadening or spectral shift of the absorption features, but the HH transition is fully bleached at the highest fluence. In contrast, in the charged film, we see significant broadening of all transient absorption features, a strong redshift of the HH transition (i.e., stimulated emission at the HH energy), and a strong reduction of all bleach amplitudes. The Δ*A* signal at ∼2.0 eV is presumably stimulated emission/state filling of free carrier transitions, since light holes will relax to the HH band, and consequently no SE of the LH exciton is expected. This bleach feature shows a significant blueshift at the highest excitation density.

**Figure 5:**
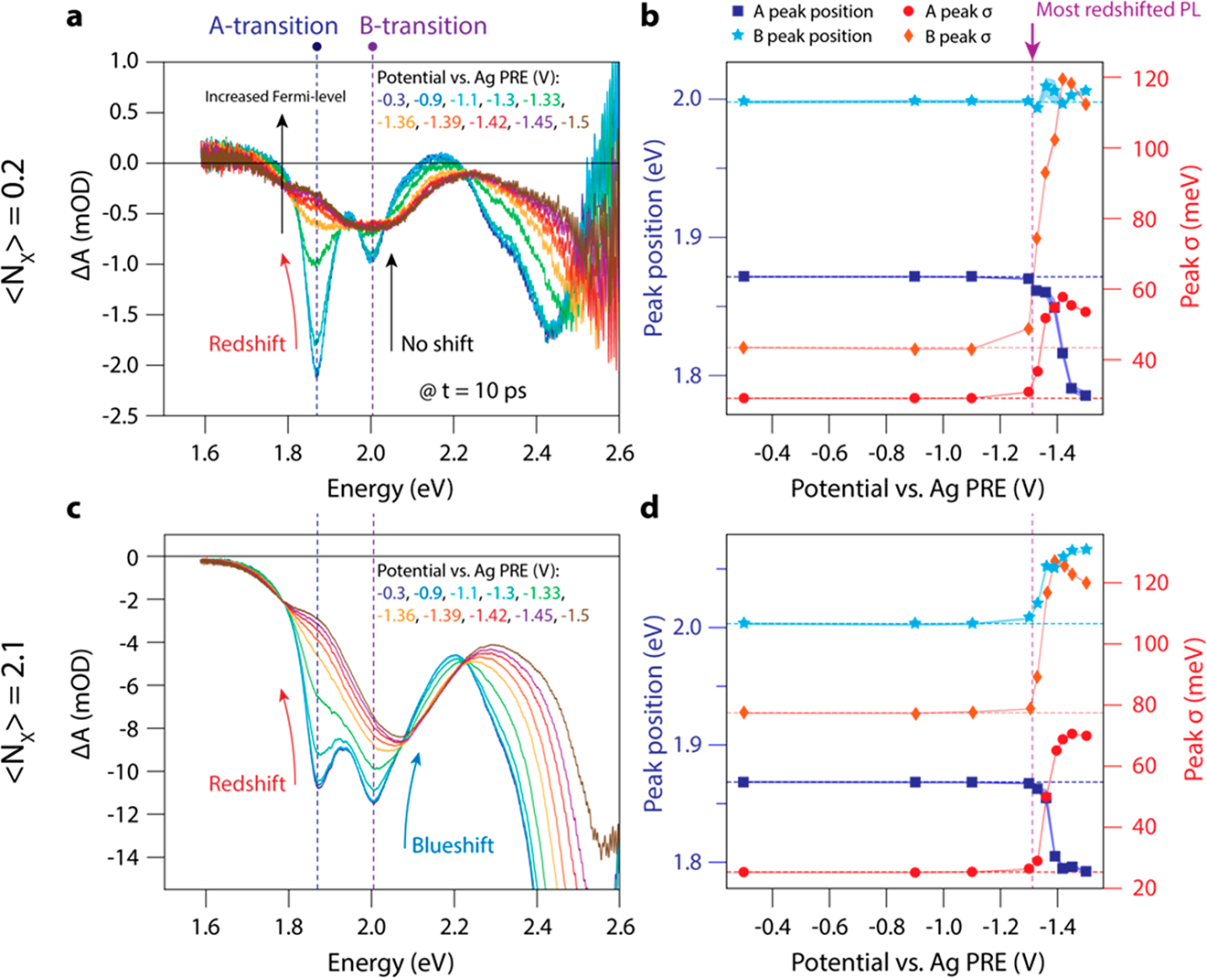
TA spectra as a function of potential and pump–probe delay time for low and high excitation density (⟨*N_X_*⟩ = 0.2 [a, b] and 2.1 [c, d]). (a) TA spectra at 10 ps pump–probe delay for various applied electrochemical potentials at low excitation density (⟨*N_X_*⟩ = 0.2). Notice how the higher energy features (related to the CdS shell) are blueshifted gradually and quenched as a function of applied potential. (b) Fitted peak position and standard deviation of the A and B transitions as a function of applied electrochemical potential. (c) TA spectra at 10 ps pump–probe delay for various applied electrochemical potentials at high excitation density (⟨*N_X_*⟩ = 2.1). (d) Fitted peak position and standard deviation of the A and B transitions as a function of applied electrochemical potential.

Figure 5a presents Δ*A* spectra for varying applied electrochemical potentials at a pump–probe delay time of 10 ps and for low (⟨*N_X_*⟩ = 0.2) excitation density. We have fitted the bleach features at the HH and LH energy (which we label “A” and “B”, respectively) with the sum of two Gaussians and plotted the resulting peak position and peak width (standard deviation) as a function of potential in Figure 5b. The A feature redshifts by 90 meV upon decreasing the potential and broadens from 29 to 58 meV. Screening of the binding energy would result in a blueshift of the HH transition, so the observed redshift must come from bandgap renormalization and/or the formation of charged excitonic species with an increased attractive interaction compared to the neutral exciton. In contrast, the Δ*A* peak at ∼2.0 eV (feature B), which we assign to overlapping LH exciton and free carrier transitions, does not show an appreciable shift in the peak position, but it broadens from 42 to 119 meV.

At higher excitation density (⟨*N_X_*⟩ = 2.1), shown in Figure 5c,d, we observe similar trends; the A transition redshifts by 70 meV and broadens from 25 to 73 meV. However, in contrast to low-excitation densities, the peak around 2.0 eV does not stay at a constant energy but blueshifts by 45 meV (next to broadening from 77 to 120 meV). The potential where the PL is most redshifted, from the SEC-PL data in Figure 2, coincides with the starting potential (purple arrow on top of Figure 5b) of the shift of bleach features A and B presented in Figure 5b,d. The position of the PL energy does not coincide with the free-carrier energy, so we suspect that the leftover PL at negative potentials comes predominantly from a fraction of NPLs in the film that is not charged. This could explain the smaller shifts (<10 meV) of the PL energy compared to the shift of the TA signals (70–100 meV).

The ultrafast spectroelectrochemistry results shown in Figures 4 and 5 show that electron injection leads to strong broadening of the absorption transitions, similar to the broadening observed in the PL upon charging (Figure 3a,e). What we certainly do not observe is the formation of sharp exciton-like bleach features in the charged NPL film, as is predicted by the scenario shown in Figure 4a. Note that SE is observed at the HH energy but that it is much broader than the HH exciton absorption in the neutral film. This confirms that severe broadening of the transitions takes place in the electron-charged NPLs in addition to state filling.

We consider that there are several causes for this broadening. First, scattering with injected electrons could cause rapid dephasing of the excitons which results in lifetime broadening of the excitonic transitions. In addition, exchange interactions between the electrochemically injected electrons and the excitations by the pump and probe pulses could induce broadening. A simple way to look at this effect is to consider that rather than forming a neutral excited species, in the charged NPL there is a broad distribution of charged excited carriers, with a resulting broad spectrum.

### Zero Threshold Optical Gain in Doped NPL Solids

We previously demonstrated that the light-amplifying properties in QD solids can be quantitatively controlled via electrochemical n doping.^9^ Since NPLs have larger material gain coefficients than QDs^13^ and show very low ASE thresholds,^10,30,46−48^ we expect a superior performance of a an n-doped film of NPLs compared to QDs for lasing applications.

**Figure 6:**
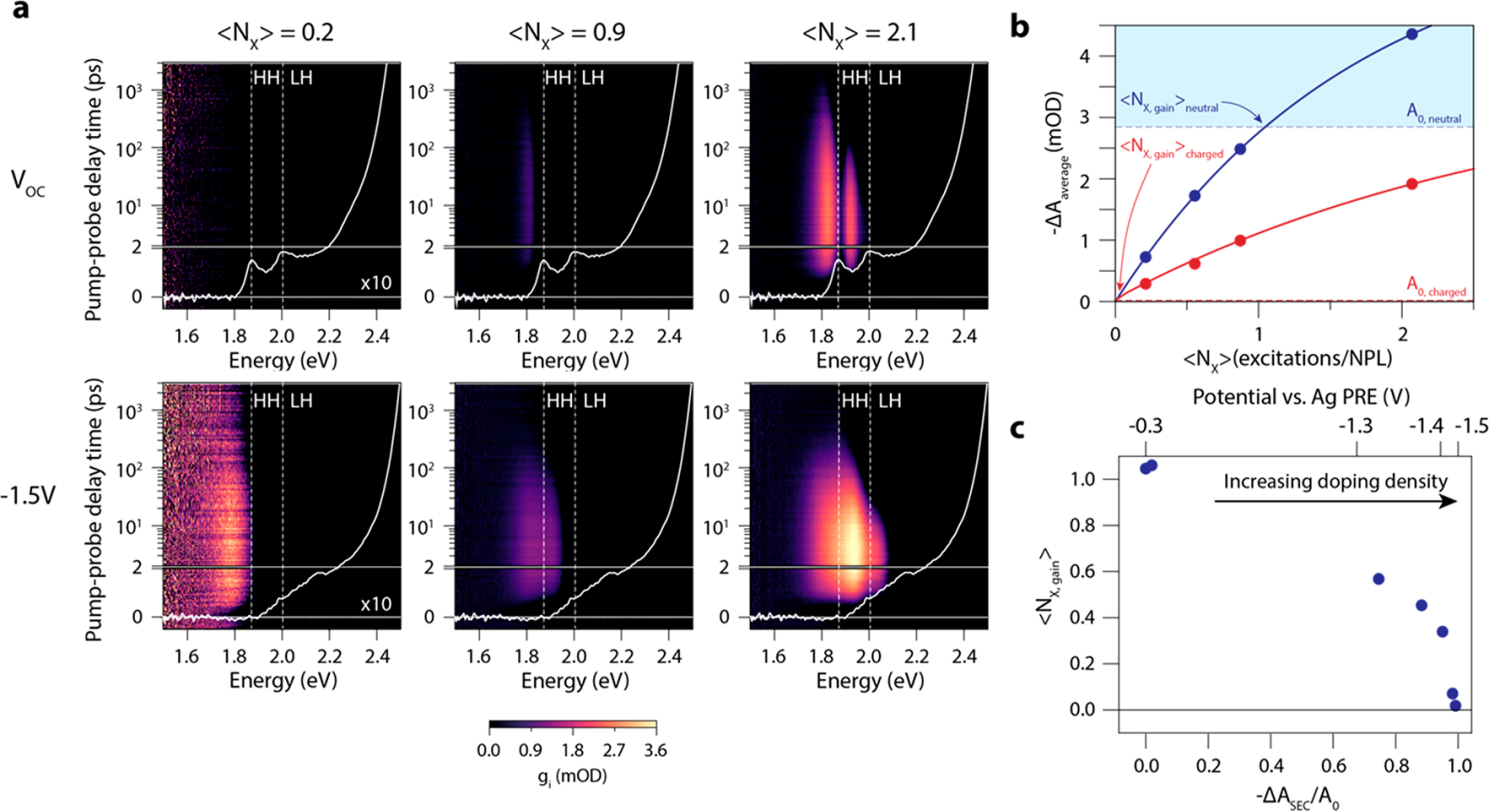
Optical gain in neutral and charged NPL solids. (a) Gain maps for different ⟨*N_X_*⟩ and doping densities (neutral, top row; n charged, bottom row). The maximum gain is achieved in between the HH and LH transition in the charged NPL film. (b) Spectrally averaged transient absorption vs excitation density. Interpolation or extrapolation is used to determine the spectrally averaged gain threshold for the undoped and doped NPL film. (c) Optical gain threshold as a function of electrochemical fractional bleach. The transient absorption is spectrally integrated over the HH transition to account for spectral shifts. The gain threshold is decreased from ⟨*N*_*X*,gain_⟩ = 1.05 to 0.02 excitons per NPL upon n charging the NPL film.

Figure 6a presents gain maps of neutral (top row) and charged (bottom row, at −1.5 V vs PRE) NPL films. Upon increasing the excitation density, the neutral film starts to exhibit optical gain around ⟨*N_X_*⟩ = 0.9 excitons per platelet, with a gain lifetime of ∼600 ps. The development of gain in the film of NPLs is very similar to that of NPLs in solution shown in Figure 2 above, albeit with narrower HH and free-carrier gain bands. Upon doping the film with electrons, shown in the bottom row of Figure 6a, we observe optical gain already at the lowest excitation density of ⟨*N_X_*⟩ = 0.2 excitons per NPL. When we further increase the fluence, the gain increases strongly. The gain features do not develop in well-defined, separated gain bands from distinct transitions but appear as broad features in the gain map.

As we did above for NPLs in solution (Figure 2e), we determine the spectrally averaged gain threshold near the band-edge transitions (averaged between 1.7 and 1.9 eV) for the neutral and charged NPL films; see Figure 6b. The gain threshold is obtained by interpolating or extrapolating the curve in Figure 6b to determine the excitation density where −Δ*A*_average_ = *A*_0,average_. We do this for decreasing potentials and plot the resulting gain threshold vs the fractional electrochemical absorption bleach Δ*A*_SEC_/*A*_0_ in Figure 6c. Upon charging the film, the spectrally integrated gain threshold is decreased from ⟨*N*_*X*,gain_⟩ = 1.05 excitons per NPL at *V*_OC_, down to ⟨*N*_*X*,gain_⟩ = 0.02 excitons per NPL (corresponding to a fluence of 170 nJ/cm^2^/pulse) at −1.5 V, demonstrating the near-complete removal of the threshold for light amplification. The spectrally integrated optical gain lifetime decreases from 1 ns in the neutral film to 500 ps in the charged NPL film.

**Figure 7:**
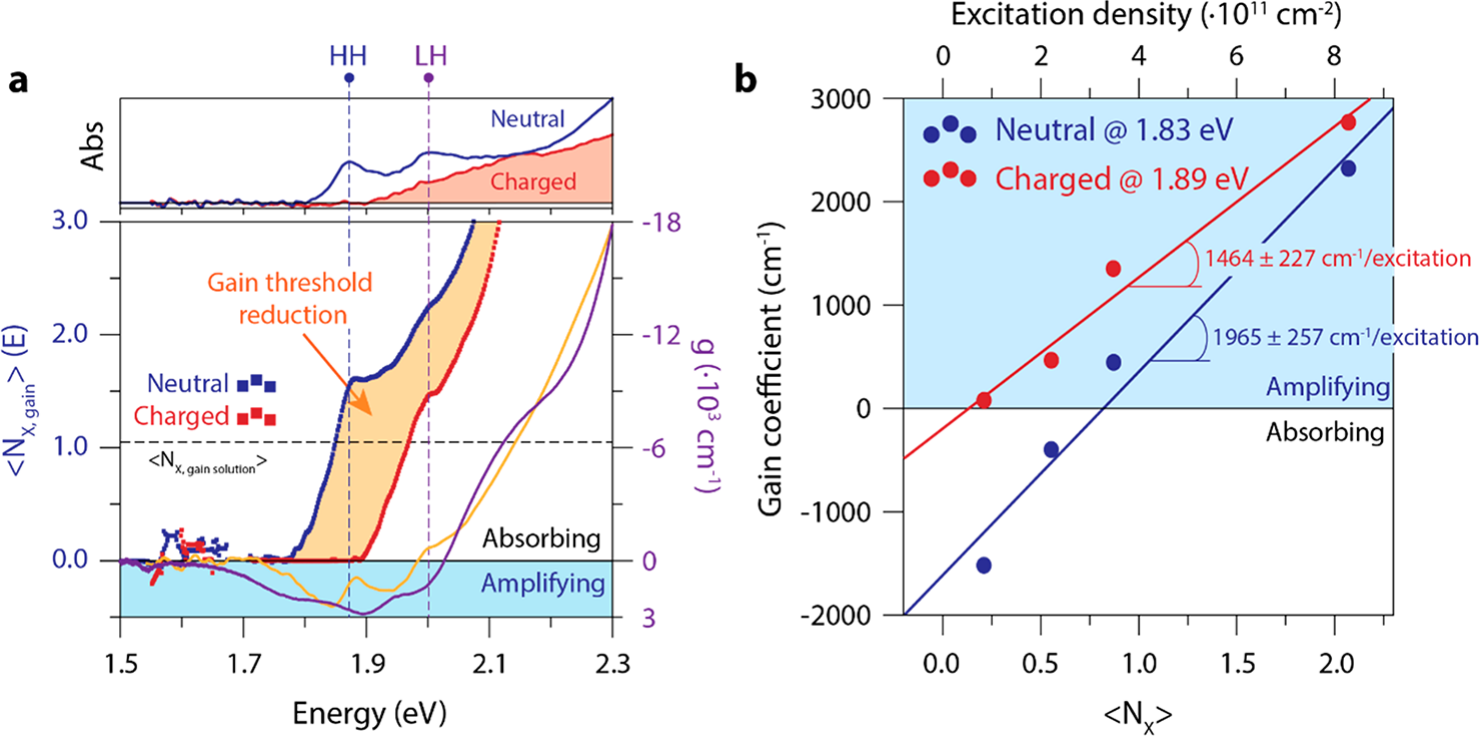
Quantifying optical gain in electrochemically doped NPL solids. (a) Gain threshold spectra for the neutral (blue) and doped (red) NPL solid. The yellow area indicates the region in which the gain threshold is significantly decreased by electrochemical doping. The top panel shows the steady-state absorption spectra of the film in the neutral and charged state. For comparison, also the gain-coefficient spectra are shown (yellow line = neutral, purple line = charged). (b) Gain coefficients as a function of excitation density at the energies with the maximum gain coefficient (1.83 eV for the neutral NPL film, 1.89 eV for the charged NPL film). To calculate the gain coefficients, we measured the film thickness to be 50.8 ± 15.5 nm and assumed a volume fraction of NPLs of 0.5 in the film.

So far, we focused on the spectrally averaged gain-threshold, since this parameter is the most relevant for discussing the photophysics. For most practical applications, the gain threshold at a single wavelength is more relevant. We plot the gain threshold spectrum in Figure 7a for neutral (blue squares) and charged (red squares) films of NPLs (i.e., the gain threshold vs energy, equivalent to the analysis of the data Figure 2f). For comparison, we also show gain coefficient spectra (yellow = neutral, purple = charged). These are obtained from the excited state absorption spectra and the film thickness *d* = 50.8 ± 15.5 nm (see Methods), as  Charging the NPLs strongly decreased the gain threshold over a wide energy range (yellow shaded area). The threshold vanishes between 1.8 and 1.9 eV, a spectral region where there is significant light amplification, as shown by the gain spectrum of the charged film (purple solid line).

In Figure 7b we plot the gain coefficients at the energies where the neutral and charged NPL film have the maximum amount of gain, 1.83 and 1.89 eV, respectively, as a function of excitation density. The gain coefficients of the charged NPLs are higher than the neutral NPLs and reach values up to 2800 cm^–1^ with no sign of saturation. The material gain per excitation, i.e., the slope of the gain coefficient vs ⟨*N_X_*⟩ curve at these energies, is 1965 ± 256 cm^–1^/excitation for the neutral NPL film and 1464 ± 227 cm^–1^/excitation for the charged NPL film. A balance between gain threshold, gain coefficient, and gain lifetime has to be sought in order to find the ideal doping density for device purposes. Compared to QDs,^9^ the NPLs have 3–4 times higher gain coefficients and are able to reach lower gain thresholds. Furthermore, the gain coefficients are higher than commonly used erbium-doped fiber amplifiers (10^–2^–10^–3^ cm^–1^) and on par with those of epitaxially grown III–V semiconductors (10^3^ cm^–1^).^49,50^ These results demonstrate the promising performance of charged NPL films as light amplifying material and highlight the possibilities of using electrochemically doped NPL solids as gain medium in low-threshold lasing devices.

## Conclusions

To summarize, we demonstrate the complex interplay of state-filling and screening of Coulomb and exchange interactions in CdSe/CdS/ZnS NPL solids, which results in zero-threshold optical gain. Both photoexcitation and electrochemical n doping of NPLs lead to a complete bleach of the excitonic transitions. We show that photoexcitation in both neutral and charged core–shell–shell NPLs leads to the formation of free carriers and that excitonic effects in these systems are weak.

Furthermore, we demonstrated that we can controllably decrease the spectrally averaged threshold for optical gain upon charging the NPL solid from 1 excitation per NPL (4 × 10^11^ cm^–2^) down to 0.02 excitations per NPL (8 × 10^9^ cm^–2^). The NPL light amplifying properties are superior to QDs, as the optical gain thresholds are lower and the gain coefficients are up to 4 times higher. Finally, we model the optical gain in our NPLs and show that there are optimal lateral sizes of the NPLs that should lead to a minimum threshold density for optical gain. Our results show that the underlying physical effects that govern gain in doped NPLs are a complicated mixture of state filling and screening effects. In short, even though their photophysics is complex, n-doped NPLs show extremely efficient optical gain which makes them highly promising materials for light amplification and lasing.

## Supporting Information Available

Description of the synthesis of the NPLs employed in this work; description of the techniques used to characterize the NPLs (TEM, steady-state absorption, PL); description of the setups used for the TA and spectroelectrochemical measurements; SEC measurements on core and core–shell NPLs; derivation of the Heisenberg model; determination of the absorption cross section using various methods (PDF)
