# Supplementary material for: Zero-Threshold Optical Gain in Electrochemically Doped Nanoplatelets and the Physics Behind It
Source: ACS Nano. 2022 Oct 18;16(11):18777–88. doi: 10.1021/acsnano.2c07519 (PMC9706803; doi:10.1021/acsnano.2c07519)
Supplement: Supplementary file 1 — nn2c07519_si_001.pdf [file nn2c07519_si_001.pdf]

Supplementary Information

for

Zero-threshold optical gain in electrochemically doped nanoplatelets and the physics behind it

Jaco J. Geuchies[†*], Robbert Dijkhuizen[†], Marijn Koel[†], Gianluca Grimaldi[†$], Indy du Fossé[†], Wiel H. Evers[†], Zeger Hens[#], Arjan J. Houtepen[†]

† Optoelectronic Materials Section, Faculty of Applied Sciences, Delft University of Technology, Van der Maasweg 9, 2629 HAZ Delft, The Netherlands

[#]Department of Chemistry and Center for Nano and Biophotonics, Ghent University, 9000 Ghent, Belgium;

* Current address: Max Planck Institute for Polymer Research, 55128 Mainz, Germany.

$ Current address: Center for Nanophotonics, AMOLF, 1098 XG Amsterdam, the Netherlands / Cavendish Laboratory, University of Cambridge, CB2 1TN Cambridge, United Kingdom.

Table of Contents

Section S1 - Methods 3

Section S2 – Determination of the HH binding energy. 14

Section S3 – Electrochemical charging of NPL films. 16

Section S4 – Temperature dependent absorption and PL of the CSS NPL film. 21

Section S5 - NPL absorption cross-section determination 22

Section S6 – Particle-in-a-box calculations of the confinement energies and thresholds for optical gain. 25

Section S7 - Heisenberg model and state-filling in NPLs 27

Section S8 – Excited state absorption, bandgap renormalization, biexciton level shifting and Stark shifts. 30

Section S9 - Additional discussion based on Schmidt-Rink et al. [5] 31

REFERENCES 33

Section S1 - Methods

Materials: 1-Octadecene (ODE) [90%, anhydrous, degassed, Sigma Aldrich], Sodium Myristate (Na(myr)) [Sigma Aldrich], Cadmium Nitrate Tetrahydrate ($Cd(NO_3)_2{\cdot}4H_2O$)[Sigma Aldrich]. Selenium powder (Se) [200 mesh, 99.999%, Sigma Aldrich], Cadmium Acetate ($Cd(Ac)_2$) [99.999%, anhydrous, Chempur] , Hexane [96%, anhydrous, TCI], Cadmium Oleate ($Cd(Oleate)_2$) [0.076M in ODE, preparation described bbelow], Methanol [99.8%, anhydrous, Sigma Aldrich], Buthanol [96.0%, anhydrous Sigma Aldrich], Oleylamine[80-90%, Sigma Aldrich], 1-Octane-Thiol [98.5%, Sigma Aldrich], Acetonitrile [99.8% anhydrous,[Sigma Aldrich], Zinc Oleate ($Zn(Oleate)_2$) [0.086M in ODE, preparation described below], Elemental Sulphur (S) [99.9995%, Sigma Aldrich], Cadmium Acetate Dihydrate ($Cd(Ac)_2{\cdot}2H_2O$) [Fluorochem], Oleic Acid [90% technical grade, degassed, Sigma Aldrich], 1,8-diaminooctane [98%, Sigma Aldrich], 1,8-octanedithiol [97%, Sigma Aldrich), ITO substrate [10x23x0.7mm, SiO_2 passivated, Prezisions glas and optik], Cadmiumdichloride ($CdCl_2$) [Sigma Aldrich], Toluene [99.8%, anhydrous, Sigma Aldrich], Ferrocene [98%, Sigma Aldrich], Lithium Perchlorate[99.9%, Sigma Aldrich]. Acetonitril was dried before use in an Innovative Technology PureSolv Micro column. All other chemicals were used as received, unless specifically mentioned.

Synthesis of bare CdSe nanoplatelets (NPLs).

For the synthesis of the bare CdSe NPLs, we first prepared Cd-myristate and Se-in-ODE precursors as sources for the NPL growth.

Cadmium precursor: Cd-myristate. $Cd(myr)_2$ is synthesized by a precipitation reaction of Na(myr) and $Cd(NO_3)_2{\cdot}4H_2O$. 1.23g (4mmol) of $Cd(NO_3)_2{\cdot}4H_2O$ was dissolved in 40ml of Methanol in a beaker. Separately, 3.13g (12.5mmol) of Na(myr) was dissolved in 250ml of Methanol in a beaker. Both beakers where continuously stirred and slightly heated (40˚C) to speed up the dissolving process. The time required to fully dissolve the Na(myr) and $Cd(NO_3)_2{\cdot}4H_2O$ was usually an hour. To prevent extensive MeOH evaporation, we placed a petridish on top of the beakers with the solutions. After preparation, the dissolved $Cd(NO_3)_2{\cdot}4H_2O$ was gently added to Na(myr) while stirring continuously. The $Cd(myr)_2$ precipitates as a fine, white powder. The entire mixture was filtrated through a Büchner funnel under vacuum. The powder was rinsed twice in the Büchner funnel with 2 times 10 mL of MeOH. The $Cd(myr)_2$ powder was then removed from the funnel and left to dry under vacuum overnight. Afterwards, the $Cd(myr)_2$ was heated to 60˚C on a hot plate for an hour inside a glovebox until all the leftover methanol was evaporated and was stored inside the box for later use.

Selenium precursor: Se-in-ODE. The Selenium precursor was made by mixing 180mg (2.28mmol) Selenium powder with 15ml of 1-Octadecene (ODE). It should be noted that Selenium powder does not dissolve in ODE, and will therefore remain as a black suspension as room temperature. The suspension should be sonicated for 5 minutes before usage.

Cd-oleate and Zn-oleate preparation. 1.32 g of Cd-(acetate)$_2$ was dissolved in 52.4g of ODE and 7.4 g of oleic acid. The mixture was placed under vacuum (10^{-1} mbar) at room temperature for 20 minutes, and heated under vacuum to 120 degrees for three hours. The Cd-oleate solution was transferred air-free into a N_2 flushed vial, and placed inside a N_2 purged glovebox for further use. Note: the Cd-oleate solution solidifies over time, which can be seen by the formation of a white sluggish gel inside the solution. Before use, the solution is heated to 50°C, until a transparent solution is formed. Usually, the formation of this sluggish gel takes a few days at room temperature. Furthermore, our Cd-oleate solutions usually had a slightly yellow tint.

The Zn-oleate was made in a similar fashion. Zn(II)-(acetate)$_2$ was mixed with 1g of OA, 1.6 mL ODE and 1.6 mL of OLAM. The oleylamine serves as a stabilizing ligand for the Zn-oleate, since this has the tendency to rapidly solidify out of solution at room temperature otherwise. The mixture was heated up in a 20 mL vial inside a nitrogen purged glovebox to 130°C and

stored there for further use. Note that the Zn-oleate solution is extremely viscous and should be handled with care when placed into a syringe.

Synthesis of the CdSe nanoplatelets. 14ml of ODE, 170mg of $Cd(myr)_2$ and 1ml of Selenium precursor and a magnetic stir bean were added to a 100mL three-neck flask. The mixture was then degassed under vacuum (10^{-1} mbar) at 50˚C while stirring continuously for an hour. After degassing, the mixture was heated to 240˚C. At 100˚C, the mixture turned transparent. At 180˚C, the mixture started turning yellow and at 200˚C the mixture turned orange. 50mg $Cd(Ac)_2$ was added when the mixture became orange, by quickly removing the nitrogen inlet and poring the Cd-acetate powder using a weighing boat, after which the N_2 inlet was placed back. After that, the particles were allowed to grow for 8 minutes and reaction mixture was then cooled to 50˚C. Lastly 12 mL of Hexane and 1 mL of 0.076M $Cd(Oleate)_2$ were added. The liquid NPL mixture was then taken out of the flask and placed in a storage vial without exposing it to air.

Purification of the CdSe nanoplatelets. The purification of the NPLs was done in two steps. First, the unreacted reagents and quantum dots were separated from the NPL solution. Second, the smaller sized nanoplatelets (460 to 480 nm luminescent NPLs) were separated from the main product (510nm NPLs). For the first purification step, a mixture of 2:1 MeOH:BuOH was

added to the NPL solution. This MeOH:BuOH mixture was added drop-wise until the solution turned turbid. The solution was then centrifuged at 3000 rpm for 15 minutes. Afterwards, the precipitate was dissolved in hexane and the supernatant discarded. This process was repeated on the precipitate for three times. For the second purification step, after the last centrifugation step, the precipitate was dissolved in 5 mL hexane and centrifuged at 4000 rpm for 30 minutes. Afterwards, the supernatant was stored in a vial and an absorption spectrum was taken, to ensure the desired product of 510 nm CdSe NPLs was obtained. The two purification steps were then repeated if impurities were still visible in the absorption spectrum.

Shell growth of bare CdSe/CdS/ZnS core-shell-shell nanoplatelets.

The CdSe/CdS/ZnS core-shell-shell synthesis used here was similar to a method by Rossinelli et al.[1] and from Kelestemur et al.[2], where one or more precursors are continuously injected through a syringe pump .The synthesis can be split in two parts:

1. Core-shell-shell synthesis from the core NPLs.

2. Purification of the core-shell-shell NPLs.

Core-shell-shell synthesis from the core nanoplatelets. Based on the synthesis here, we obtain 6 monolayers of CdS and 2 monolayers of ZnS on each side of the CdSe NPLs (confirmed by checking the absorption peak positions after CdS shell growth to reported values in literature from Ithurria et al.[3]). To start, 2 mL CdSe core NPLs stock solution, 1.3mL Cd-oleate (0.076M) and 2.5mL ODE were added to a 50mL three-neck flask. The mixture was degassed for 40 minutes at room temperature and then degassed for 20 minutes at 80˚C. After degassing, 1mL of oleylamine was added and the temperature was increased to 300˚C. Starting at 180˚C a solution of 28 μL (0.16mmol) 1-octanethiol dissolved in 4mL ODE was injected at a rate of 1.5mL/h by a syringe pump. After two hours, a solution of 0.04 mmol Zn-oleate in 2 mL ODE was injected at 1.5ml/h, simultaneous with the octanethiol. After completion of the addition of the precursors, the solution was kept at 300°C for 30 more minutes, and afterwards cooled down to room temperature by removing the heating and cooling with an airgun. Lastly, 2.5mL hexane was added to the synthesis product. The synthesized CdSe/CdS/ZnS core-shell-shell NPL solution was taken out of the flask and stored in a nitrogen purged vial.

Core-shell-shell nanoplatelet purification. The purification was done in three steps. In the first step, the NPL solution was centrifuged at 4000 rpm for 30 minutes. In the second step, the precipitate was re-dispersed in 5mL hexane and a few droplets of oleylamine. The re-dispersed

NPL solution was then again centrifuged at 4000 rpm for 30 minutes. In the third step a 2:1 MeOH:BuOH mixture was added to the supernatant of the mixture and was then once again centrifuged at 4000 rpm for 30 minutes. The last two steps were repeated twice for further purification. Afterwards, the precipitate was re-dispersed in 5 mL hexane and stored in a vial inside a nitrogen purged glovebox. Absorption and PL spectra were recorded and further purification was done by repeating the last step if necessary, based on these spectra.

QDs-on-ITO film preparation by dipcoating. We used the core-shell-shell NPL solution as obtained by the procedure above. Before dipcoating, the ITO slides were cleaned by sonication in isopropanol and rinsing with ethanol and acetone, followed by drying with an airgun. The slides were placed inside a UV-Ozone cleaner for 30 minutes prior to dipcoating, to increase the wetting of the NPL solution on the ITO. The dipcoating was performed using a dipcoater from Nima Technology, while our glovebox was set on 'purge mode' to ensure that the solvents are extracted from the box efficiently by the continuous flow of N_2 gas. The ITO slides were consecutively dipped for 30 seconds in a solution of the colloidal NPLs (1), a solution of 0.1M 1,8-octanedithiol in MeOH (2) and pure MeOH (3) to remove an excess of unbound ligands. In between the consecutive dipping steps, the solvent (hexane, MeOH) was allowed to evaporate for 30 seconds. These steps were repeated at least 20 times to ensure the build-up of a decently thick film. Roughly 1/3rd of the entire substrate was left uncoated (and cleaned with a cotton

swab and ethanol later) to ensure good contact with the electrodes in our electrochemical experiments (see below).

Steady state absorption and photoluminescence measurements. Absorption spectra were measured on a double-beam PerkinElmer Lambda 1050 UV/Vis spectrometer; in case of the NPL films on ITO, the sample was measured inside an integrating sphere and an empty ITO was measured separately for background correction. Photoluminescence spectra were recorded on an Edinburgh Instruments FLS980 spectrofluorimeter equipped with double grating monochromators for both excitation and emission paths and a 450 W Xenon lamp as an excitation source.

Transmission Electron Microscopy (TEM). TEM images were acquired using a JEOL JEM-1400 plus TEM microscope operating at 120 kV. Samples for TEM imaging were prepared by dropcasting a dilute solution of NPLs onto a Formvar and carbon coated copper (400-mesh) TEM grid.

fs-Transient Absorption (TA) spectroscopy. fs-TA measurements are performed on solutions of the CdSe(/CdS/ZnS) NPLs in hexane or toluene, loaded inside an air-tight cuvet inside a nitrogen purged glovebox. A Yb-KGW oscillator (Light Conversion, Pharos SP) is used to produce 180 fs photon pulses with a wavelength of 1028 nm and at a frequency of 5 kHz. The pump beam is obtained by sending the fundamental beam through an Optical Parametric Amplifier (OPA) equipped with a second harmonic

module (Light Conversion, Orpheus), performing non-linear frequency mixing and producing an output beam whose wavelength can be tuned in the 310-1330 nm window. A small fraction of the fundamental beam power is used to produce a broadband probe spectrum (480-1600 nm), by supercontinuum generation in a sapphire crystal. The pump beam is transmitted through a mechanical chopper operating at 2.5 kHz, allowing one in every two pump pulses to be transmitted. Pump and probe beam overlap at the sample position with a small angle (roughly 8°), and with a relative time delay controlled by an automated delay-stage. After transmission through the sample, the pump beam is dumped while the probe is collected at a detector (Ultrafast Systems, Helios). During the experiments, we make sure the pump and probe beam have orthogonal polarizations (i.e. one of them is vertically polarized, the other horizontally), to reduce the influence of pump scattering into our detector. The differential absorbance is obtained via $\Delta A = log(I_{on}/I_{off})$, where I is the probe light incident on the detector with either pump on or pump off. TA data are corrected for probe-chirp via a polynomial correction to the coherent artifact. Pump photon fluence was estimated by measuring the power with a thermopile sensor (Coherent, PS19Q) and obtaining the beamshape with a beamprofiler.

We also measure transient reflection (TR) spectra to obtain the true change in absorption in transient transmission experiments.

Photoluminescence quantum yield (PLQY) measurements. We measured the PLQY of the NPL dispersions with respect to a Rhodamine 101 solution in ethanol. The PLQY was calculated using the following equation;

$$PLQY = PLQY_{Rhodamine\ 101} \frac{I^{PL}_{QD\ solution}}{I^{PL}_{Rhodamine\ 101}} \frac{f_{Rhodamine\ 101}}{f_{QD\ solution}} \left(\frac{n_{hexane}}{n_{ethanol}}\right)^2$$

Where $PLQY_{rhodamine\ 101}$ is set to be 95%, I^{PL} is the intensity of the photoluminescence signal of either the QD solution or the Rhodamine 101 solution, $n_{hexane/ethanol}$ is the refractive index of hexane or ethanol at 530 nm (1.377 and 1.3630) and f_x is the fraction of absorbed light of species x, calculated as $f_x = 1 - 10^{-OD_x}$, where OD_x is the optical density of the solution containing either the QDs or the Rhodamine 101. We determined the PLQY of the CdSe/6CdS/2ZnS core-shell-shell NPLs to be 62%.

Spectroelectrochemical (SEC) measurements. The SEC measurements were all performed in a N_2 purged glovebox. As an electrolyte, we used an 0.1 M $LiClO_4$ solution in acetonitrile, which was dried with an Innovative Technology PureSolv Micro column. The QD film was immersed in the electrolyte solution, together with a Ag wire pseudoreference electrode and a Pt sheet counter electrode. The potential of the NC film on ITO was controlled with a PGSTAT128N Autolab potentiostat. Changes in the absorption or PL of the NC film as a function of applied potential were recorded simultaneously with a cyclic voltammogram with a fiber-based UV-VIS spectrometer (USB2000, Ocean Optics). For the film, the measurements were started at the open-circuit potential (V_{OC} = -0.3V w.r.t. Ag wire, i.e. -

0.75V vs. Fc/Fc^{+})), while scanning with a rate of 10 mV/s. Unless stated otherwise, all potentials are given w.r.t. the Ag pseudoreference. For SEC measurements combined with fsTA, ultrafast spectroelectrochemistry, we loaded the samples inside a nitrogen purged glovebox into a leak-tight sample holder.

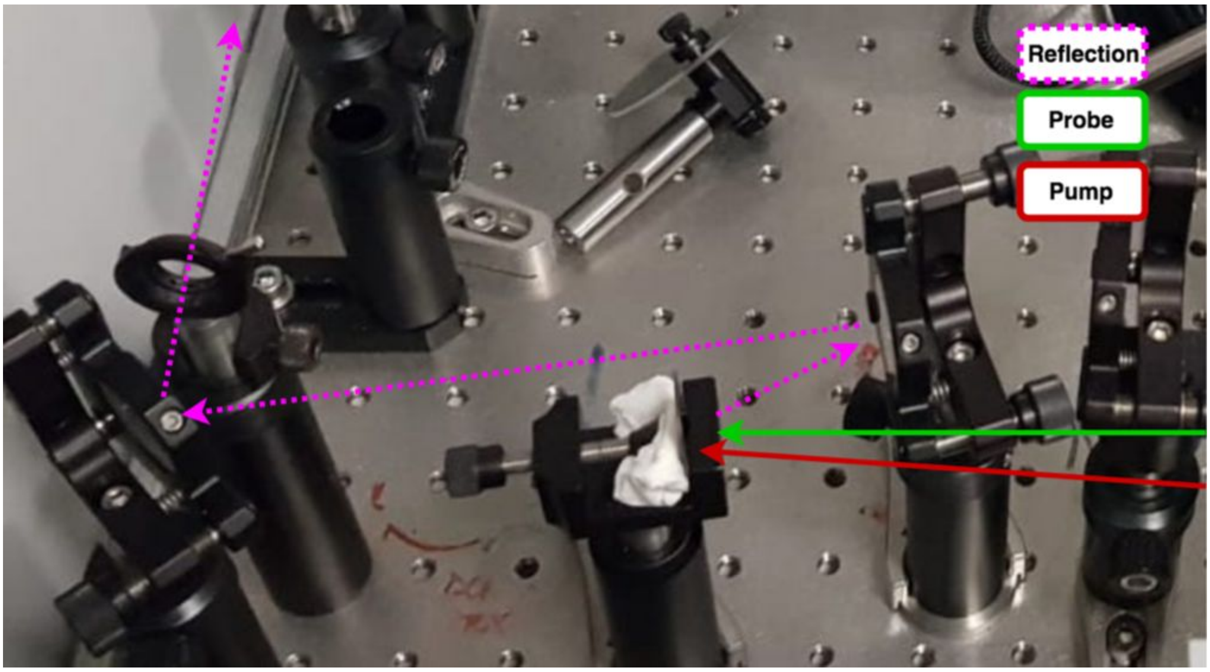

Figure S1: Alignment of pump and probe for the transient reflection experiments. The transmitted probe beam is blocked after the sample (not shown in picture). Also here, pump and probe polarizations are orthogonal to each other.

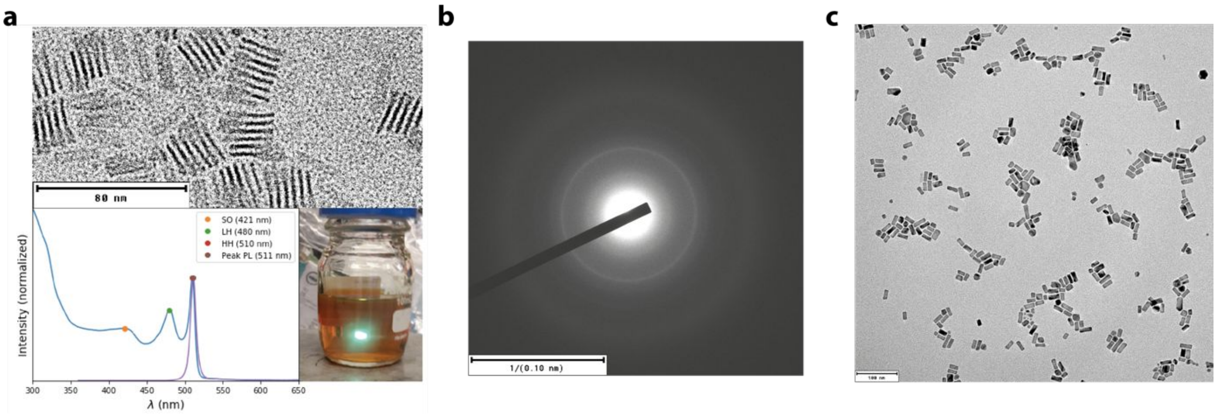

Figure S2: Optical and structural characterization of the core CdSe NPLs and the CdSe/6CdS/2ZnS core-shell-shell NPLs used throughout this study. (a) Absorption and PL of the core CdSe NPLs, absorbing at 510 nm (bottom). Representative TEM image of the cores (top) and photograph (bottom right) of the NPL stock solution. **(b)** Electron diffraction pattern of the core CdSe NPLs, indicating a zinc-blende crystal structure (note the strong contrast with a wurtzite crystal structure [prevalent in many quantum-dots], due to the missing two sets of triplet peaks). **(c)** Overview TEM image of the core-shell-shell NPLs, where we grew 6 monolayers of CdS and 2 monolayers of ZnS over the core NPLs. These NPLs were used throughout the main part of the manuscript.

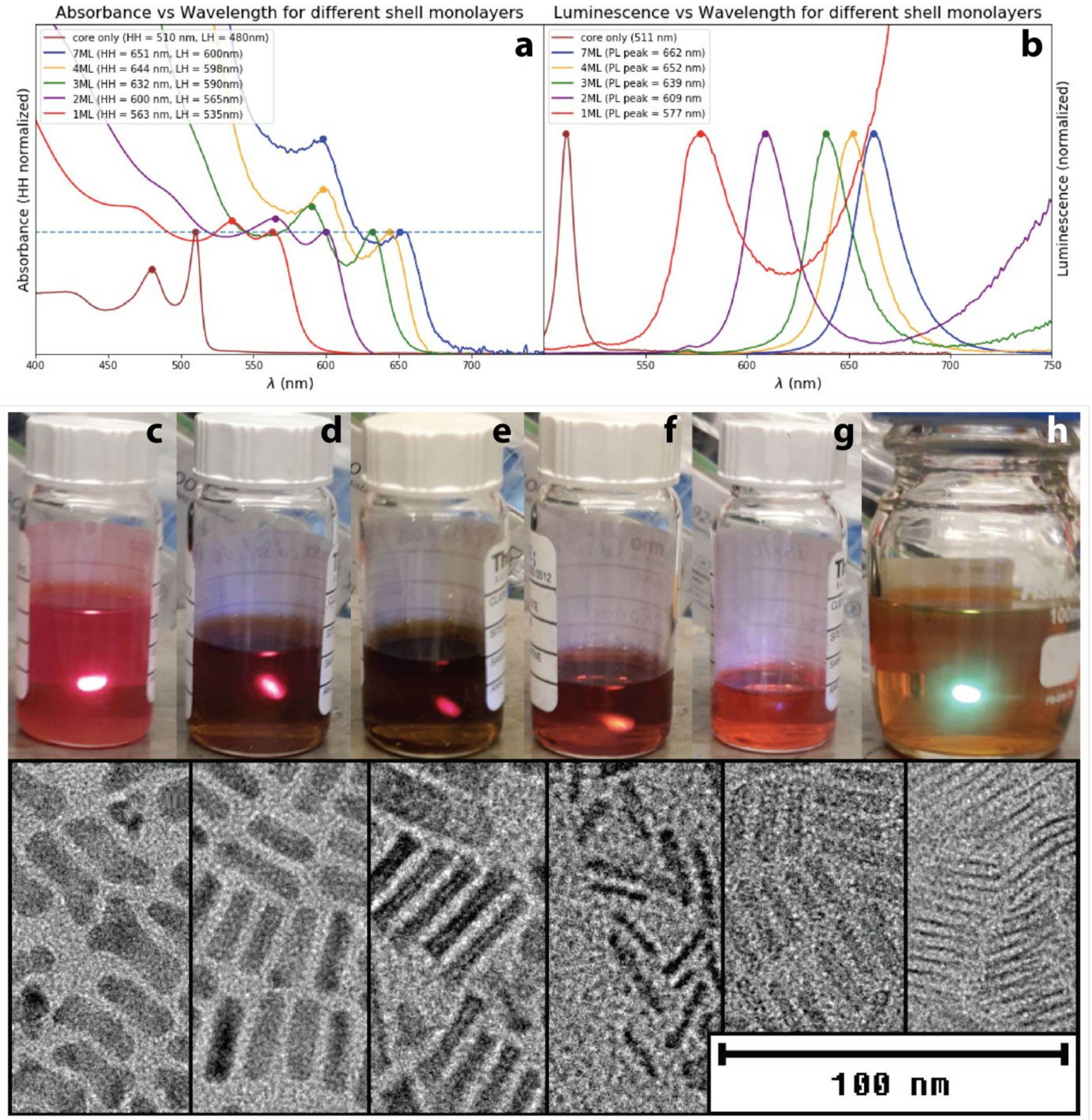

Figure S3: Optical and structural characterization of CdSe/CdS NPLs grown by continuous injection. (a) Absorption and **(b)** PL of the core-shell CdSe/CdS NPLs with different shell thicknesses. The shell thickness was assigned according to work by Ithurria et al. [3]. **(c-g)** Photographs of the colloidal solutions (top) and TEM images of the CdSe/*n*CdS core-shell NPLs, with n = 7,4,3,2,1 going from left to right respectively, and the core-only CdSe nanoplatelets on the right in **(h)**.

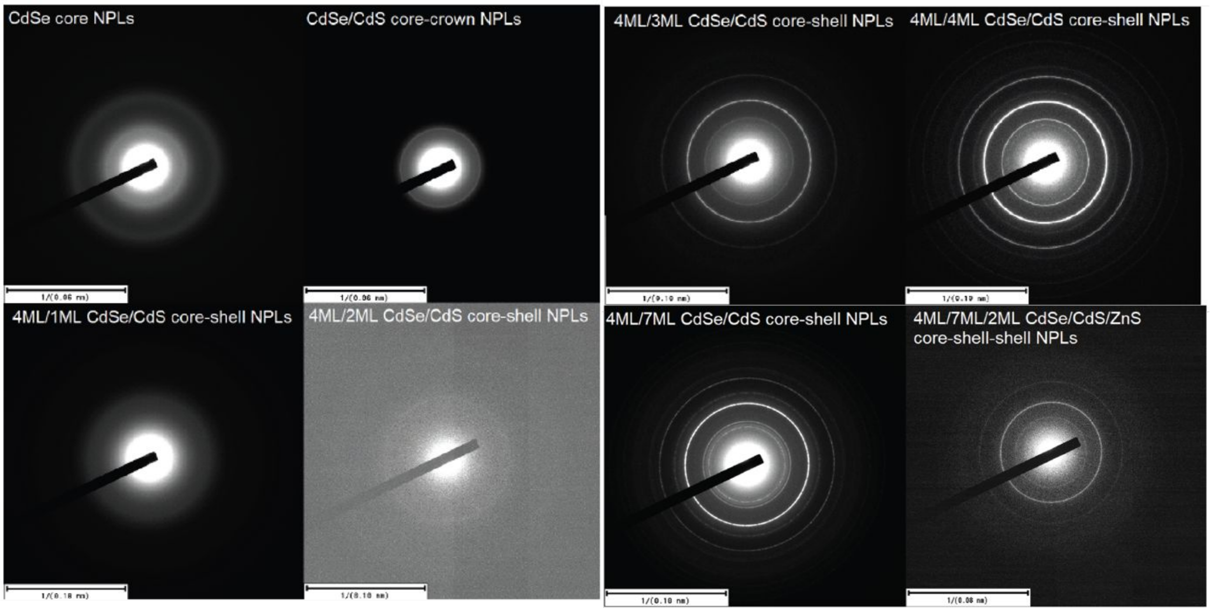

Figure S4: Electron diffraction on all types of NPLs. The core-only, core-shell, and core-shell-shell NPLs all keep a zinc-blende crystal structure. Number of monolayers (ML) is indicated in the top of each electron diffractogram.

Section S2 – Determination of the HH binding energy.

We make a crude estimate of the exciton binding energy vs. the shell thickness by fitting the absorption spectra with an adjusted Elliot-model[4].

$$A(E) = p_X(E) + C(E)$$

Where A(E) is the absorption as a function of energy, p_X the absorption of a quantum well exciton with asymmetric broadening η due to localization:

$$p_X(E) = \frac{1}{2\eta}\left[\text{erf}\left(\frac{E-E_0}{\gamma} - \frac{\gamma}{2\eta}\right) + 1\right] exp\left(\frac{\gamma^2}{4\eta^2} - \frac{E-E_0}{\eta}\right)$$

and C(E) the absorption profile for free-carrier transitions:

$$C(E) = \frac{A_c}{2}\text{erf}\left(\frac{E-E_0-E_{b,X}}{\gamma_C}\right)$$

Here, E_0 and $E_{b,X}$ are the exciton energy and exciton binding energy, respectively. 'Erf' is the error function. The total absorption becomes the sum of both LH and HH contributions:

$$A_{total}(E) = A_{HH} + A_{LH}$$

This model decomposes the absorption spectrum into an excitonic and free-carrier contribution part. We include both the HH and LH exciton and free carrier contributions, and fit the region around the band-edge. For shell thicknesses larger than 3 CdS monolayers, it is very hard to observe the onset of the free-carrier absorption in the absorption spectrum due to the increasing CdS shell absorption. Therefore, we use the 0, 1, 2 and 3 monolayer fits and extrapolate it to

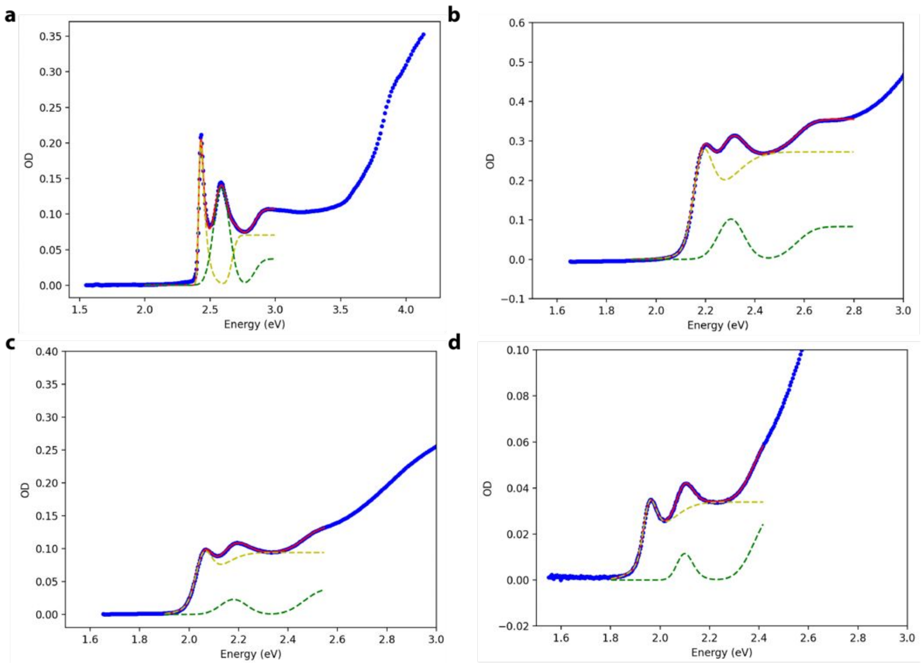

the 6 monolayer thickness using a heuristic fit (shown in Fig. 1 of the main text). We note that this is a very rough estimation of the exciton binding energy for the NPLs used throughout this study.

Figure S5: Fits of the absorption spectra with an adjusted Elliot model for quantum-wells to the absorption of the NPLs in solution for bare, 1ML, 2ML and 3ML (a-d) respectively. The yellow dashed line is the HH contribution, the green dashed line the LH contribution and the red line

the sum of the two. The distinction from free-carrier absorption (broadened errorfunction) and exciton contribution (Lorentzian) can be clearly seen. The range of the data used for fitting is displayed by the plotted range of the fits data itself.

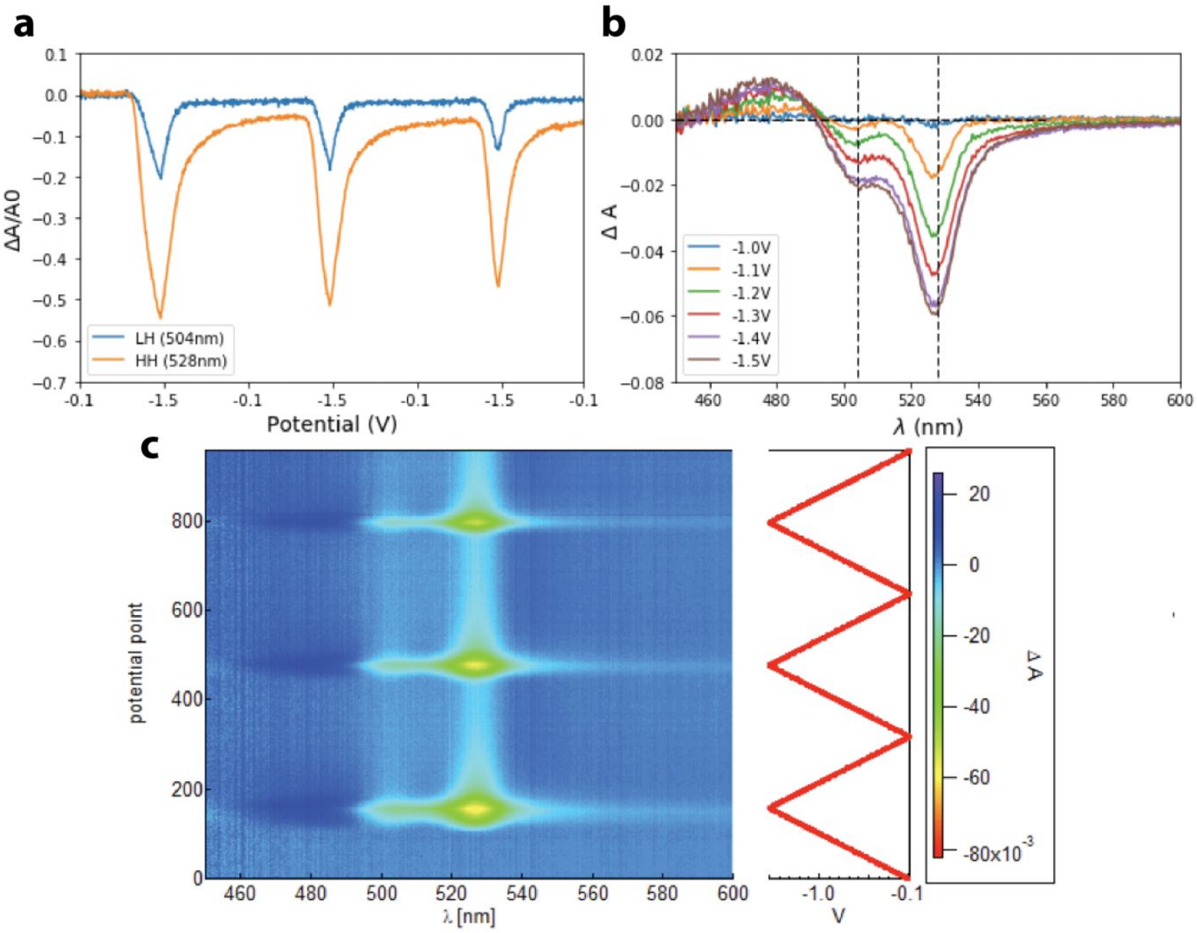

Section S3 – Electrochemical charging of NPL films.

Figure S6: Spectroelectrochemistry on a film of core-only CdSe NPLs. We did not observe any photoluminescence from the film. **(a)** Fractional bleach (DA/A_0) versus applied electrochemical

potential for the HH and LH transitions. We did not obtain a full bleach (DA/A_0). The LH bleach is likely convoluted with a shifted feature. **(b)** Spectral cuts at different applied electrochemical potentials. **(c)** Two-dimensional absorbance SEC map. Charge extraction is much slower than charge injection; the bleach features disappeared after the measurement, when we forced the system to stay at the open-circuit potential for 30 minutes. The sub-bandgap 'bleach' feature is likely a change in reflectivity of the film.

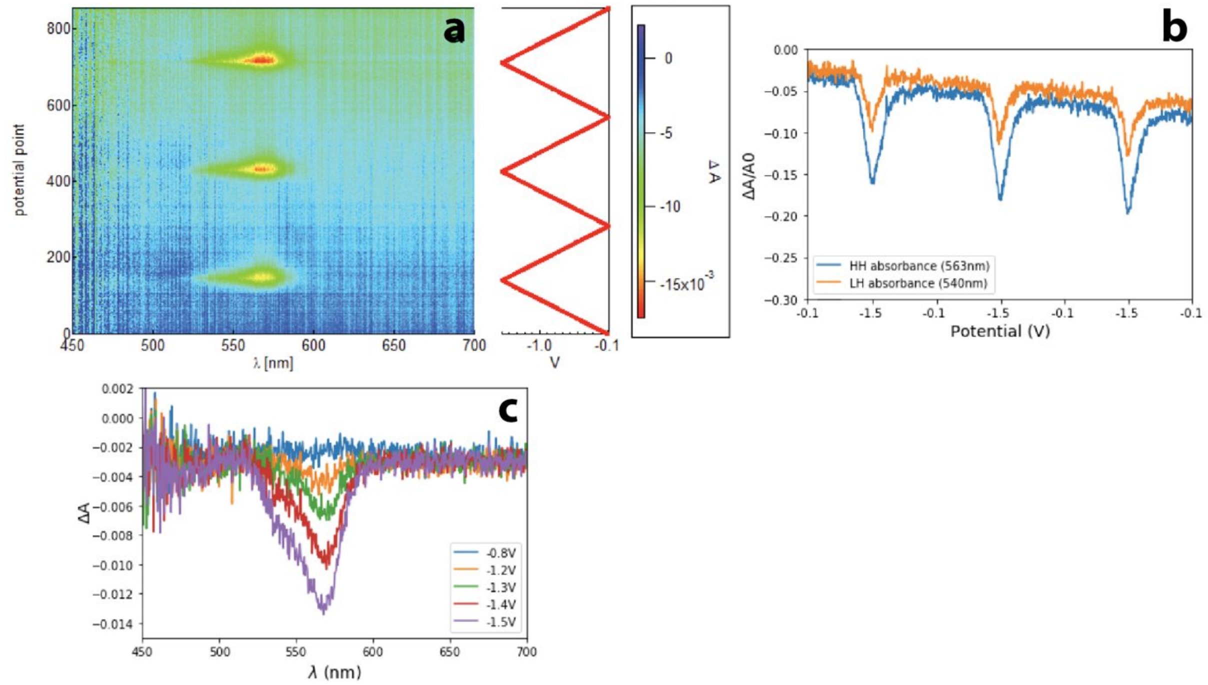

Figure S7: Spectroelectrochemistry on a film of core-shell CdSe/1CdS NPLs. We did not observe any photoluminescence from the film. **(a)** Two-dimensional absorbance SEC map. **(b)**

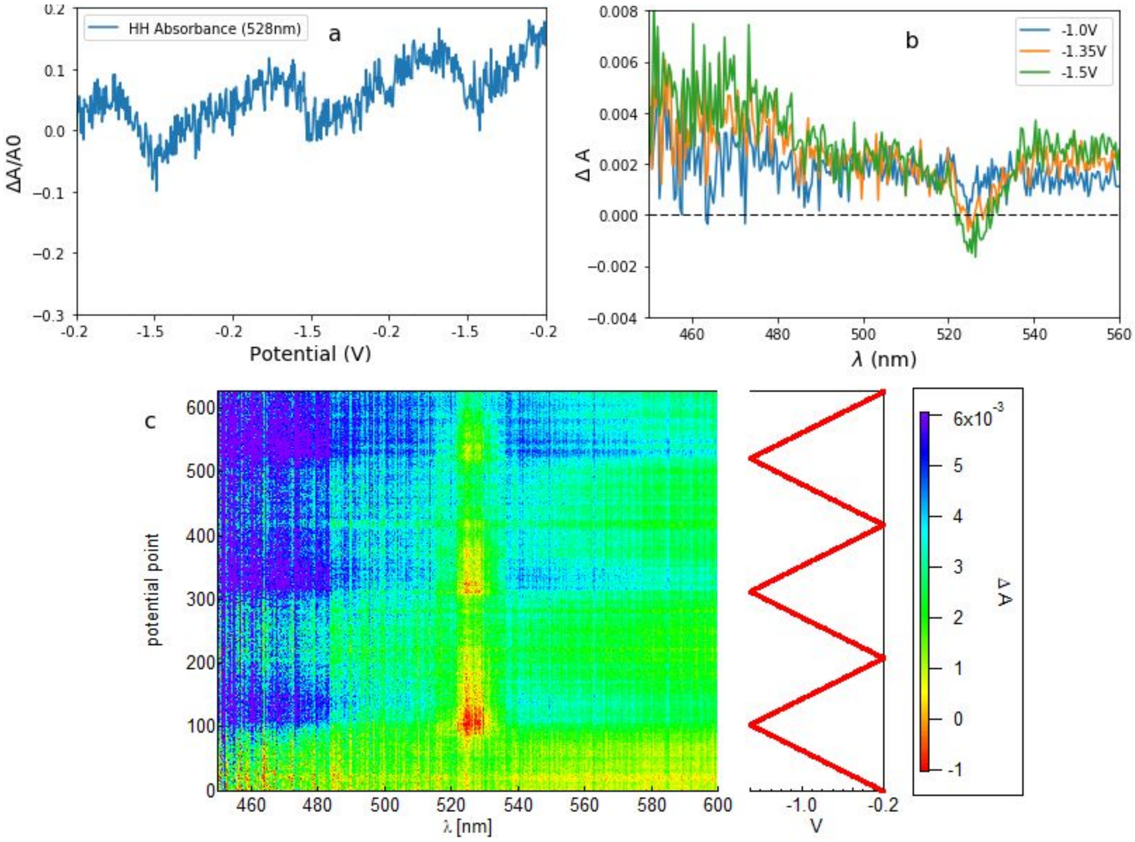

Fractional bleach vs. applied electrochemical potential for the LH and HH transitions. **(c)** DA spectra at different applied electrochemical potentials.

Figure S8: Spectroelectrochemistry on a film of core-crown CdSe/CdS NPLs. We did not observe any photoluminescence from the film. **(a)** Fractional bleach as a function of applied potential. **(b)** DA spectra at different applied electrochemical potentials. **(c)** Two-dimensional absorbance SEC map.

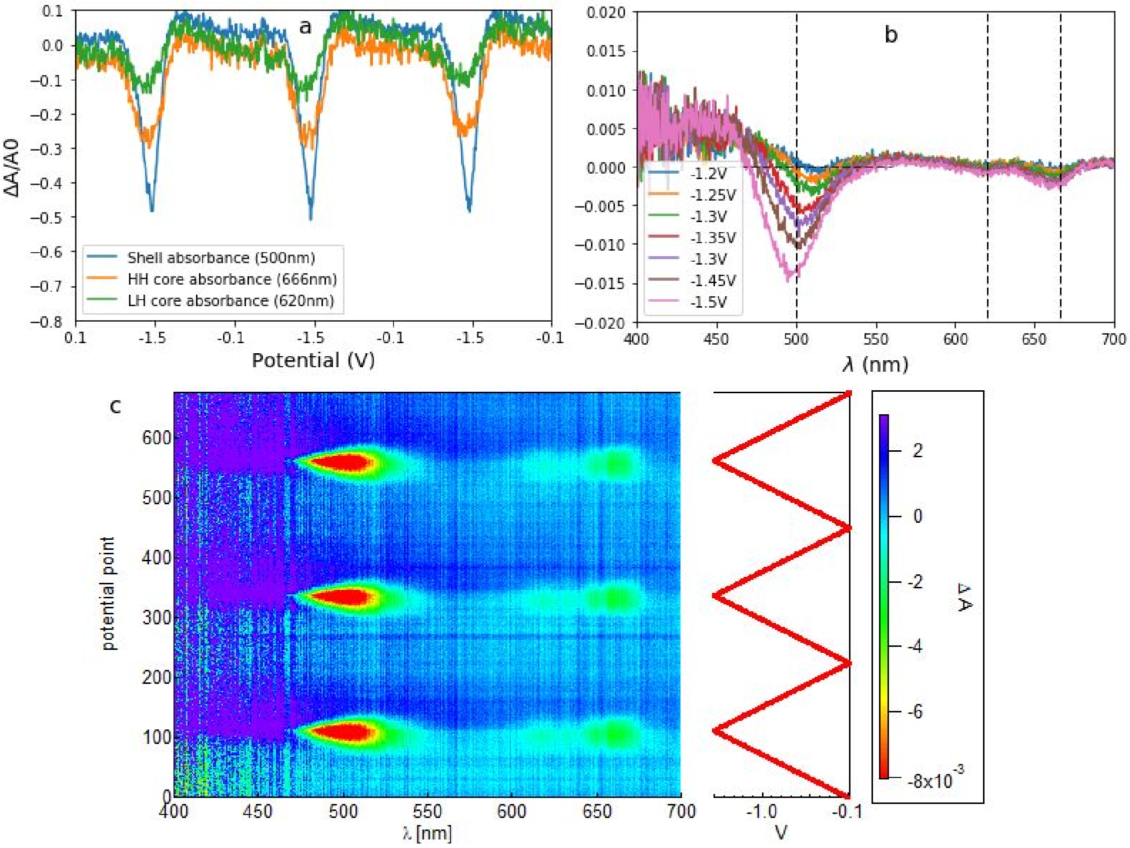

Figure S9: Representative absorption spectroelectrochemistry on a film of core-shell CdSe/6CdS NPLs. (a) Fractional bleach as a function of applied potential. **(b)** DA spectra at

different applied electrochemical potentials. (c) Two-dimensional absorbance SEC map. Note that thinner CdS shells led to less reversible charging and discharging of the NPL films.

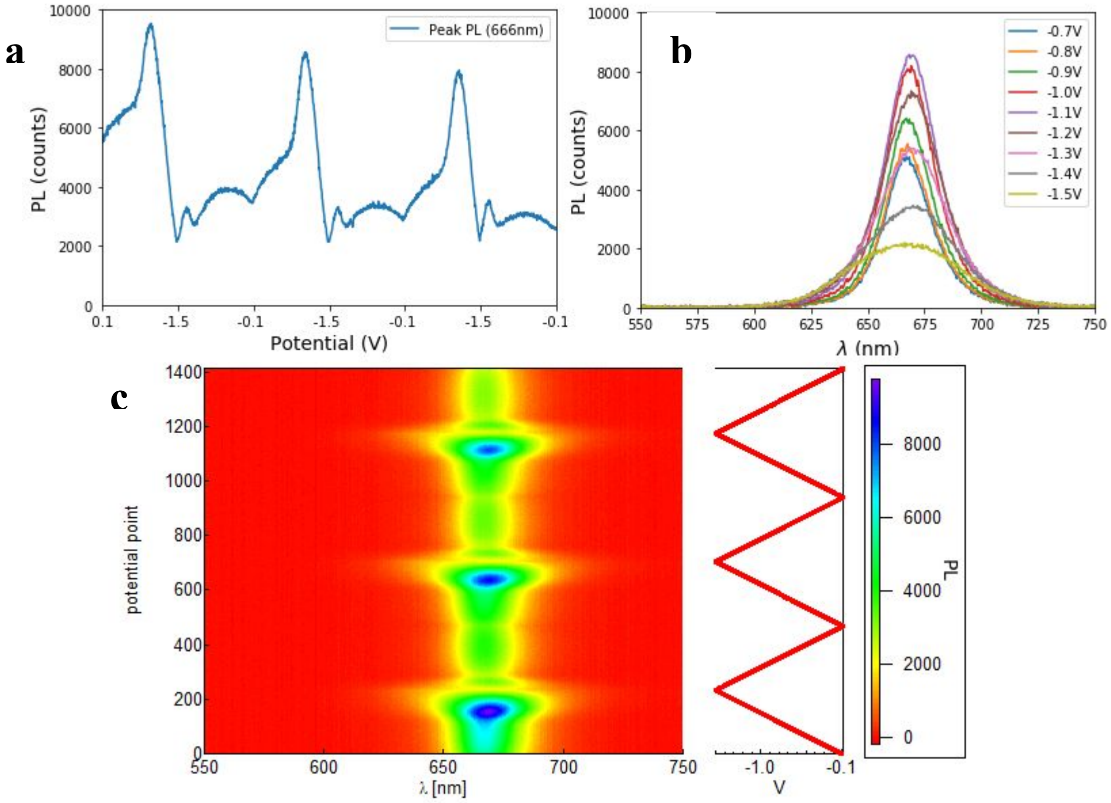

Figure S10: Representative PL spectroelectrochemistry on a film of core-shell CdSe/6CdS NPLs. (a) Integrated photoluminescence intensity as a function of applied potential. **(b)** PL spectra at different applied electrochemical potentials. **(c)** Two-dimensional PL SEC map. Note that thinner CdS shells lead to less reversible charging and discharging of the NPL films.

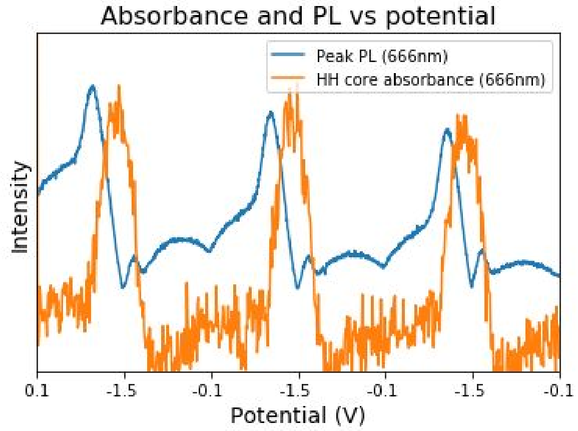

Figure S11: Comparison between absorption bleach onset and PL quenching versus applied electrochemical potential in the CdSe/6CdS core-shell NPL film.

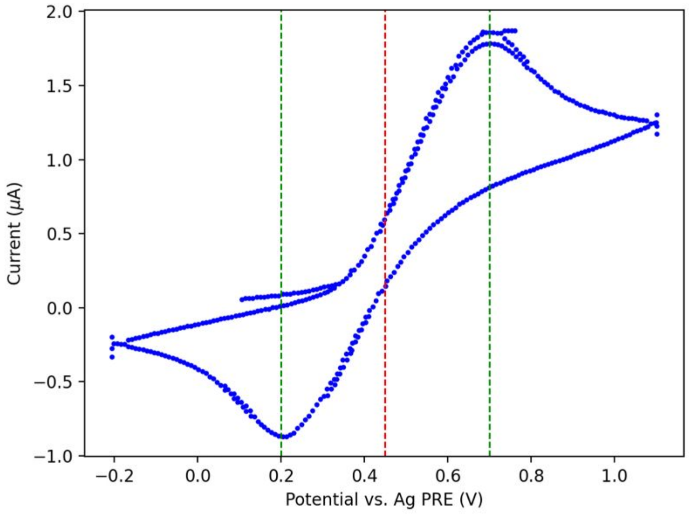

Figure S12: Calibration of the Ag pseudo-reference electrode with the Fc/Fc$^+$ redox couple before the electrochemical TA experiments. The green lines indicate the positions of the oxidation and reduction waves (+0.7 V and +0.2V respectively), and its' potential is given by the red line at +0.45 V. Since Fc/Fc$^+$ lies -4.7 eV below vacuum, the Ag PRE lies at -4.35 eV (or + 4.35 V) below vacuum. No significant shift of the reference potential was found after the SEC-TA experiments.

Section S4 – Temperature dependent absorption and PL of the CSS NPL film.

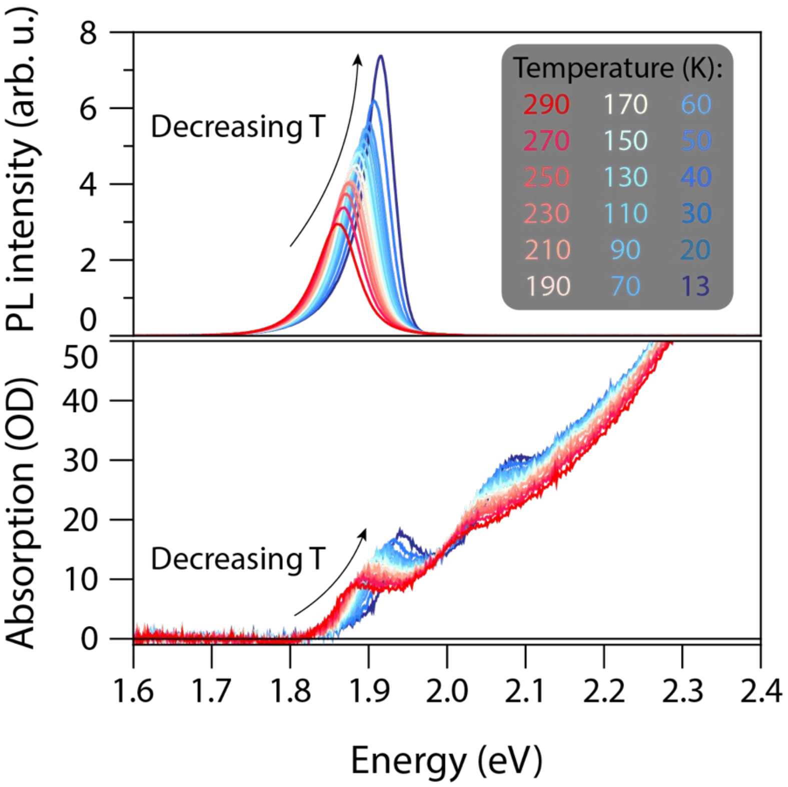

Figure S13: Temperature dependent absorption and photoluminescence of a film of core-shell-shell NPLs.

Section S5 - NPL absorption cross-section determination

We determine the NPL absorption cross section using three methods:

1. **Poisson statistics of Auger recombination.** The magnitude of the bleach between 1-3 ns pump-probe delay time is smaller than directly after photoexcitation, but nonzero, meaning that there still is a finite population of nanocrystals which have excitons in there. The amplitude of the absorption bleach scales with the number of excitons present, and can be estimated via;

$$|\Delta A_{1-3\,ns}| \propto 1 - P_0 = 1 - e^{-\langle N \rangle}$$

Where $|\Delta A_{1\text{-}3ns}|$ is the magnitude of the bleach between 1-3 nanoseconds, P_0 is the Poisson probability of finding zero excitons and is the average exciton population per nanocrystal. In turn, $\langle N \rangle = \sigma J_0$, with σ being the absorption cross section at the excitation wavelength, and J_0 the incoming photon fluence. By fitting the data to the above equation, we obtain an absorption cross section at 400 nm of $5.6 \pm 0.2 \cdot 10^{-14}$ cm^2, which we use to calculate per photon fluence used in the TA experiments. For this analysis, we also correct the incoming photon fluence J_0 for absorption throughout the solution:

$$J'_0 = \frac{1 - e^{-\alpha L}}{\alpha L} J_0$$

with the average of the photon fluence across the solution J'_0 length and α the absorption coefficient at the excitation wavelength. The term α·L equals A·ln(10), with A the optical density at 400 nm.

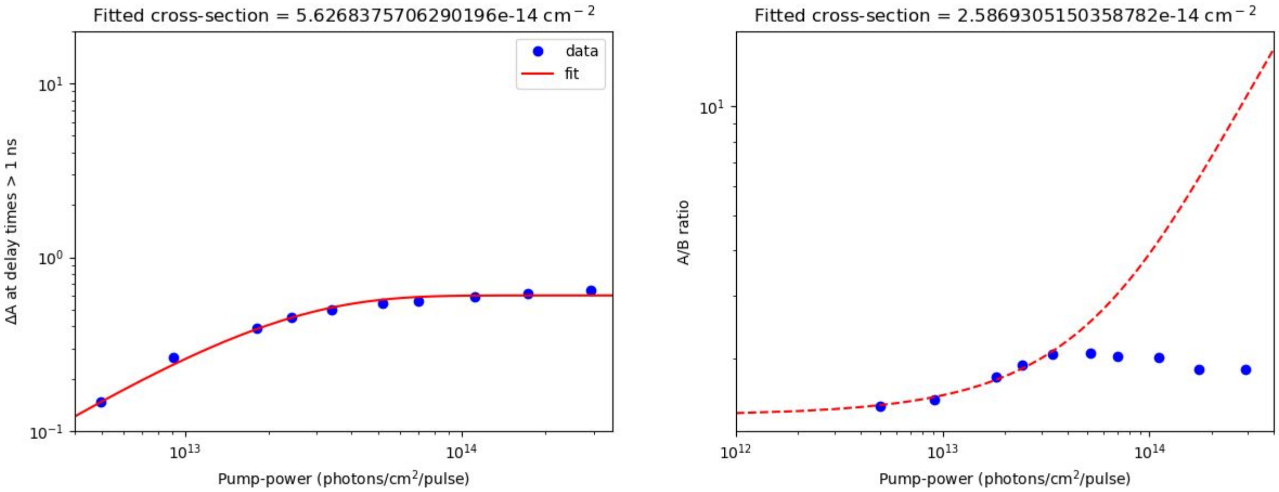

Figure S14: NPL cross section determination using Poisson statistics of Auger recombination (left) and the 'A-over-B-ratio' (right). The data in the left panel was fitted with equation SXX and resulted in an absorption cross-section at 400 nm of 5.6±0.2·10^{-14} cm^2. The data in the right panel was fitted with equation SXX – over the first 5 datapoints - and resulted in an absorption-cross section at 400 nm of 2.6±0.4·10^{-14} cm^2.

2. **'A-over-B ratio'.** The A/B ratio is used frequently in carrier multiplication experiments to determine quantum yields. The A-over-B ratio is defined as:

$$\frac{A}{B} = \frac{\Delta A_{t=0}}{\Delta A_{1-3ns}} = \frac{\sigma J_0'}{1 - e^{-\sigma J_0'}}$$

We determined the A/B ratio for various fluences, and fit with the above equation to obtain a cross section of $2.6 \pm 0.4 \cdot 10^{-14}$ cm^2. Note that we only fit the initial 5 datapoints, since the equation does not take into account the saturation of the early-time bleach at higher fluences. The result is shown in Figure SXX. The cross section determined with this method is 2.2 times lower than determined via method 1.

3. **Calculation of the absorption cross-section based on known dielectric constants.** We estimate the absorption cross section of our CSS NPLs based on known dielectric functions of the bulk materials, obtained from https://refractiveindex.info/. We find values for k at 320 nm.

CdSe: Ninomiya and Adachi 1995: Cubic. $k = 1.1052$.
CdS: Ninomiya and Adachi 1995: $k = 0.65143$.
ZnS: Ozaki and Adachi 1994: Cubic ZnS. $k = 0.3484$.

From k we calculate the intrinsic absorption coefficient μ, as $\mu = \frac{4\pi k}{\lambda}$. Using the number of CdS (5) and ZnS (2) monolayers we predict from the synthesis on top of our NPLs, we calculate the volume weighted intrinsic absorption coefficient (weighing each intrinsic absorption coefficient by the volume of that material present), and obtain a total intrinsic absorption coefficient at 320 nm of $1.85 \cdot 10^5 \mathrm{cm}^{-1}$. From the volume of the CSS NPLs V_{NPL} (1418.1 nm^3), we calculate the absorption cross section at our pump wavelength (400 nm) as

$$\sigma_{400\,nm} = \mu_{320\,nm} V_{NPL} \frac{A_{400\,nm}}{A_{320\,nm}}$$

where we use the ratio between the absorption at 400 nm and 320 nm to obtain the absorption cross section at our pump wavelength – $9.4 \cdot 10^{-14}$ cm^2. This value is 1.7 times higher than the value obtained via method 1.

Since method 2 produces a lower and method 3 produces a higher cross section, we decide to use the cross section from method 1 throughout the paper, which is roughly the average from all three methods.

Section S6 – Particle-in-a-box calculations of the confinement energies and thresholds for optical gain.

For a particle in a 2D box, the confinement energy is given by:

$$E_{confinement} = \frac{h^2}{8m^*}\left(\left[\frac{n_x}{L_x}\right]^2 + \left[\frac{n_y}{L_y}\right]^2 + \left[\frac{n_z}{L_z}\right]^2\right)$$

With h being Planck's constant, m^* the effective mass of the particle (0.13 for the electrons and 0.3 for holes in CdSe), $n_{x,y,z}$ the quantum numbers in the x, y and z directions and $L_{x,y,z,}$ the lengths of the box in the x, y, and z directions. For the CSS NPLs we used (L_x = 24.9 nm, L_y = 9.9 nm), we calculate a total confinement energy in the NPL plane (we ignore the thickness), i.e. the sum of the electron and hole confinement energies, of 49 meV. For the difference in energy from the first to second quantized level in the longest direction (n_x, n_y = 1,1 to n_x, n_y = 2,1) we find an energy of 20 meV.

Relevant for determining the onset of gain is which energy levels are occupied at room temperature. Occupation of higher levels starts to play a role when the splitting between the subsequent quantum confined energy levels is of the order of kT. For the z-direction (the thickness of the NPLs) the splitting between n_z = 1 and n_z = 2 is much larger than kT, so we ignore this contribution.

Threshold for optical gain

For a 2D bulk sheet of CdSe, there should be a continuous density-of-states in the x and y directions. We calculate the threshold for optical gain by calculating at which concentration of electrons and holes the quasi-Fermi level is equal to the conduction band and valence band, respectively. For bulk semiconductors this is standard, since for all levels below the quasi-Fermi level the occupation is larger than 0.5, and hence there is population inversion.

For the 2D sheets, we calculate the energy levels based on a simple particle-in-a-box calculation, again without excitonic effects. We again calculate the density at which the quasi-Fermi levels are equal to the first empty and filled states (i.e. the LUMO and HOMO levels in the NPLs).

Modelling optical gain in NPLs: optimal NPL lateral size for lowest threshold excitation fluence

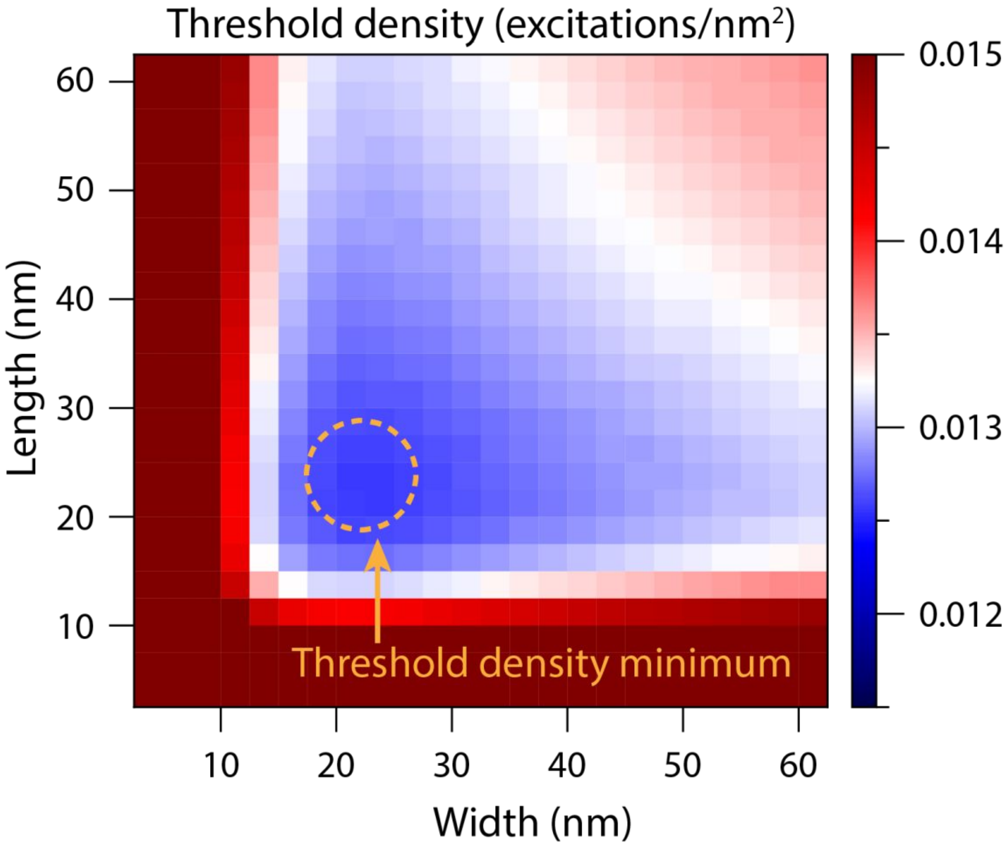

Figure S15: Modelling the optical gain in NPLs with a particle-in-a-box model. Threshold density for optical gain from a state-filling model. There is an optimum in threshold density for a NPL lateral size of roughly 23 by 23 nm.

Section S7 - Heisenberg model and state-filling in NPLs

We find that we can fully bleach the lowest excitonic transition in CdSe/CdSe/ZnS NPLs by electrochemically injecting electrons. We can also fully bleach the transition by optical

excitation. However, there is a large difference in TA signals for neutral and charged NPLs: neutral NPLs show sharp bleach features, charged ones show extreme broadening.

The question is how to explain the absorption bleach features (both electrochemical and optical): do they result from state filling or from screening of the e-h Coulomb interaction (Mott transition). Our tentative conclusion is that at low excitation densities (<N> smaller than 1) we can explain most things by state filling alone, but we cannot exclude both effects happen at higher excitation densities. Screening results in a strong decrease of the exciton binding energy, resulting in fast exciton dissociation and corresponding lifetime broadening. This is especially important for the electrochemically charged case. The observed broadening corresponds to an exciton (or rather, charged exciton) lifetime of ~40 fs.

This does not mean state filling is not important for electrochemical charging. The question is how to model that, since we fill up electron states, but we observe a bleach of the exciton transition. Since excitons and electrons are described by different wavefunctions, the exciton wavefunction should be expended in electron states, or vice versa. Here we use a rather hand waving estimate based on the Heisenberg uncertainty principle to estimate up to what energy (or up to what Fermi-level) the $n_z = 1$ band needs to be filled to fully bleach the 1S exciton.

Model based on Heisenberg's uncertainty relation

Starting from Heisenberg:

$$\Delta x\,\Delta k \geq \frac{1}{2}$$

Where Δx and Δk are the (Gaussian) uncertainties in position and momentum. For the uncertainty in position, we make an assumption that this is roughly the exciton Bohr radius: $\Delta x \approx a_B$, changing the uncertainty relation into $a_B\,\Delta k \geq \frac{1}{2}$.

Now we can express the Bohr radius in a material by the Bohr radius of the 1S hydrogen wavefunction $a_B = \frac{a_0 \varepsilon_R}{\mu}$, where ε_R is the real part of the static dielectric constant and μ the reduced effective mass, which can be expressed as

$$\frac{1}{\mu} = \frac{1}{m_e^*} + \frac{1}{m_h^*} \approx \frac{1}{m_e^*}$$

Here we use that the effective mass of the electron (0.13) is three times lower than that of the hole (0.3) in zinc-blende CdSe.

Furthermore, we can express in terms of a set of constants;

$$a_0 = \frac{4\pi\varepsilon_0 \hbar^2}{m_0 e^2}$$

We can write an expression for the uncertainty of the total energy in terms of the uncertainty in in-plane wavenumber and

$$\Delta E_{tot} = \frac{\hbar^2 \Delta k_x^2}{2\, m_e^*\ m_0} + \frac{\hbar^2 \Delta k_y^2}{2\, m_e^*\ m_0} = \frac{\hbar^2 \Delta k^2}{m_e^*\ m_0}$$

Using the above equations, we find that

$$\Delta E_{tot} = \frac{\hbar^2 \Delta k^2}{m_e^*\ m_0} = \frac{\hbar^2}{4\, m_e^*\ m_0\, a_B^2} = \frac{\hbar^2\, m_e^{*\,2}}{4\, m_e^*\ m_0\, a_0^2\, \varepsilon_R^2} = \frac{\hbar^2\, m_e^*}{4\, m_0\, a_0^2\, \varepsilon_R^2}$$

When we use accepted values for the bulk exciton Bohr radius in CdSe, 5.4 nm, an effective mass of the electron of 0.13, and the static dielectric constant in CdSe of 10.2, we for ΔE_{tot} of about 31 meV.

Discussion

We calculated a value of roughly 31 meV from our model, compared to the roughly 140 mV necessary to fully bleach the HH exciton transition. This would suggest that state filling should more quickly bleach the exciton transition than observed.

A reason why this value is too small, is that the dielectric constant should be roughly 2 times smaller in a two-dimensional system. This would make the estimated energy ~40 meV. Even

when we vary this around a bit, we still get an estimate that is smaller than the 140 mV we measure experimentally.

Furthermore, as discussed throughout the main text, we also mention that the obtained width of the bleach vs. potential curve can be larger due to drops of the electrochemical potential (change in potential does not correspond 1:1 to change in Fermi level in the NPL film).

Section S8 – Excited state absorption, bandgap renormalization, biexciton level shifting and Stark shifts.

The optical transitions in semiconductors often shift in energy upon photoexcitation. Several effects can cause shifting of these transitions, but the underlying origins of these shifts are often used synonymously throughout literature, which can be confusing:

- A biexciton shift is the total energy difference between the photon energy needed to create a first exciton and the photon energy needed to create the second exciton, and it hence contains all physical effects that change the transition energy.

  This shift can be due to purely electrostatic effects, i.e. the net Coulomb interaction between all four carriers in a biexciton is not exactly twice the net Coulomb interaction between the electron and hole in a single-exciton. This Coulomb effect is often referred to as Stark shifting.

- Bandgap renormalization is the change of the electron/hole free carrier bands due to optical excitation as a result of the electron-hole exchange energy.

The sum of bandgap renormalization and Coulomb interactions (a.k.a. Stark shifting) is what is observed in experiments, and it is in practice very hard to disentangle.

Section S9 - Additional discussion based on Schmidt-Rink et al. [5]

Optical excitation or electronic doping leads to the occupation of some free carrier states that are now no longer available for the formation of new excitons. This leads to a decrease of the exciton absorption, an effect that is called state filling. In the case of a large exciton binding energy, and a correspondingly small Bohr radius, the number of k-states that contributes to the exciton wavefunction is large, and the bleach of a single free carrier (occupying a state near k=0) is small. For a smaller binding energy this bleach will be larger.

Considering only the effect of state-filling on the absorption bleach Schmitt-Rink et al. derived that the saturation density N_S, the excitation density where the exciton absorption is fully bleached, is equal to[5]

$$\frac{1}{N_S} = 8\pi a_{X,2D}^2 \tag{a1}$$

Eq. a1 is valid, according to Schmitt-Rink, for above resonant excitation (as we use in our experiments), which results directly in the formation of free electrons and holes rather than excitons and for the case when $kT \ll E_{B,HH}$. Entering the exciton Bohr radius of 3.2 nm, derived from the experimentally determined exciton binding energy, this gives a saturation density of $N_S = 3.9 \cdot 10^{11}$ cm^{-2}, *exactly* in line with the experimentally determined gain threshold. For

excitons (e.g. formed by resonant excitation and before exciton dissociation takes place), the expected saturation density is given by[5]

$$\frac{1}{N_S} = \frac{32}{7}\pi a_{2D}^2 \qquad \text{(a2)}$$

which gives a saturation density of $N_S = 6.8 \cdot 10^{11}$ cm^{-2}. The saturation density for free-carrier excitation matches our experimentally observed saturation density better than the saturation density for excitons. Note that this does not mean that the observed optical gain stems from free-carrier species, since the probe pulse in the experiment still generates excitons around 1.9 and 2.0 eV.

The above arguments based on state filling explain why the exciton bleaches in our NPLs, without invoking screening of Coulomb or exchange interactions. As the saturation density in our NPLs corresponds to 1 exciton/NPL, we cannot rule out that at higher excitation densities, Coulomb and exchange effects do play a crucial role. Indeed, severe broadening of the excited state absorption is observed in NPLs where the excitation density is high [$\langle N \rangle \gg 1$, Figure 2(d) of the main text]. Again, this indicates that the core-shell-shell NPLs act as particle-in-a-box-like systems.

REFERENCES

(1) Rossinelli, A. A.; Rojo, H.; Mule, A. S.; Aellen, M.; Cocina, A.; De Leo, E.; Schäublin, R.; Norris, D. J. Compositional Grading for Efficient and Narrowband Emission in CdSe-Based Core/Shell Nanoplatelets. *Chem. Mater.* **2019**, *31* (22), 9567–9578. https://doi.org/10.1021/acs.chemmater.9b04220.

(2) Kelestemur, Y.; Shynkarenko, Y.; Anni, M.; Yakunin, S.; De Giorgi, M. L.; Kovalenko, M. V. Colloidal CdSe Quantum Wells with Graded Shell Composition for Low-Threshold Amplified Spontaneous Emission and Highly Efficient Electroluminescence. *ACS Nano* **2019**, *13* (12), 13899–13909. https://doi.org/10.1021/acsnano.9b05313.

(3) Ithurria, S.; Talapin, D. V. Colloidal Atomic Layer Deposition (c-ALD) Using Self-Limiting Reactions at Nanocrystal Surface Coupled to Phase Transfer between Polar and Nonpolar Media. *J. Am. Chem. Soc.* **2012**, *134* (45), 18585–18590. https://doi.org/10.1021/ja308088d.

(4) Tomar, R.; Kulkarni, A.; Chen, K.; Singh, S.; Van Thourhout, D.; Hodgkiss, J. M.; Siebbeles, L. D. A.; Hens, Z.; Geiregat, P. Charge Carrier Cooling Bottleneck Opens Up Nonexcitonic Gain Mechanisms in Colloidal CdSe Quantum Wells. *J. Phys. Chem. C* **2019**, *123* (14), 9640–9650. https://doi.org/10.1021/acs.jpcc.9b02085.

(5) Schmitt-Rink, S.; Chemla, D. S.; Miller, D. A. B. Theory of Transient Excitonic Optical Nonlinearities in Semiconductor Quantum-Well Structures. *Phys. Rev. B* **1985**, *32* (10), 6601–6609. https://doi.org/10.1103/PhysRevB.32.6601.
